# Supplementary material for: Diterpenoids with Potent Anti-Psoriasis Activity from Euphorbia helioscopia L
Source: Molecules. 2024 Aug 29;29(17):4104. doi: 10.3390/molecules29174104 (PMC11397010; doi:10.3390/molecules29174104)
Supplement: Supplementary file 1 [file molecules-29-04104-s001.zip › molecules-3149183-supplementary.pdf]

Supplementary Materials

Diterpenoids with Potent Anti-Psoriasis Activity from *Euphorbia helioscopia* L.

Zhen-Zhu Zhao[1], Xu-Bo Liang[1], Hong-Juan He[2], Gui-Min Xue[1], Yan-Jun Sun[1], Hui Chen[1], Yin-Sheng Zhao[1], Li-Na Bian[1], Wei-Sheng Feng [1,*], Xiao-Ke Zheng[1,*]

[1]School of Pharmacy, Henan University of Chinese Medicine, Zhengzhou 450046, China

[2] Academy of Chinese Medical Sciences, Henan University of Chinese Medicine, Zhengzhou 450046, China

Corresponding Authors

*Tel/fax: 86-27-67842267. E-mail: fwsh@hactcm.edu.cn (W. -S. Feng)

*Tel/fax: 86-27-67842267. E-mail: zhengxk.2006@163.com (X. -K. Zheng)

Table of Contents

1. Spectroscopic data 4
Table S1 ^{1}H (500 MHz) and ^{13}C NMR (125 MHz) data of compounds **13**, **17** and **20** ($CDCl_3$, δ in ppm, J in Hz)..4
Figure S1 1H NMR spectrum of **1** (500 MHz, $CDCl_3$) 5
Figure S2 ^{13}C and DEPT 135 NMR spectra of **1** (125 MHz, $CDCl_3$) 5
Figure S3 HSQC spectrum of **1** 6
Figure S4 ^{1}H-^{1}H COSY spectrum of **1**...6
Figure S5. HMBC spectrum of **1** 7
Figure S6 ROESY spectrum of **1** 7
Figure S7 HRESIMS report of **1**...8
Figure S8 ^{1}H NMR spectrum of **2** (500 MHz,)...9
Figure S9 ^{13}C and DEPT 135 NMR spectra of **2** (125 MHz, $CDCl_3$) 9
Figure S10 HSQC spectrum of **2** 10
Figure S11 ^{1}H-^{1}H COSY spectrum of **2**...10
Figure S12 HMBC spectrum of **2** 11
Figure S13 ROESY spectrum of **2**..11
Figure S14 HRESIMS report of **2**...12
Figure S15 ^{1}H NMR spectrum of **3** (500 MHz, $CDCl_3$)..13
Figure S16 ^{13}C NMR spectrum of **3** (125 MHz, $CDCl_3$)..13
Figure S17 HSQC spectrum of **3** 14
Figure S18 ^{1}H-^{1}H COSY spectrum of **3**...14
Figure S19 HMBC spectrum of **3** 15
Figure S20 ROESY spectrum of **3**..15
Figure S21 HRESIMS report of **3**...16
Figure S22 ^{1}H NMR spectrum of **4** (500 MHz, $CDCl_3$)..17
Figure S23 ^{13}C NMR spectra of **4** (125 MHz, $CDCl_3$) 17
Figure S24. HSQC spectrum of **4** 18
Figure S25 ^{1}H-^{1}H COSY spectrum of **4**...18
Figure S26 HMBC spectrum of **4** 19
Figure S27 ROESY spectrum of **4**..19
Figure S28 HRESIMS report of **4**...20
Figure S29 ^{1}H NMR spectrum of **5** (500 MHz, $CDCl_3$)..21
Figure S30 ^{13}C spectrum of **5** (125 MHz, $CDCl_3$)...21
Figure S31 HSQC spectrum of **5** 22
Figure S32 ^{1}H-^{1}H COSY spectrum of **5**...22
Figure S33 HMBC spectrum of **5** 23
Figure S34 ROESY spectrum of **5**..23
Figure S35 HRESIMS report of **5**...24
Figure S36 ^{1}H NMR spectrum of **6** (500 MHz, $CDCl_3$)..24
Figure S37 ^{13}C NMR spectrum of **6** (125 MHz, $CDCl_3$)..25

Figure S38 HSQC spectrum of **6** 26
Figure S39 ^{1}H-^{1}H COSY spectrum of **6** 26
Figure S40 HMBC spectrum of **6** 27
Figure S41 ROESY spectrum of **6** 27
Figure S42 HRESIMS report of **6** 28
Figure S43 ^{1}H NMR spectrum of **7** (500 MHz, $CDCl_3$) 29
Figure S44 ^{13}C NMR spectrum of **7** (125 MHz, $CDCl_3$) 29
Figure S45 HSQC spectrum of **7** 30
Figure S46 ^{1}H-^{1}H COSY spectrum of **7** 30
Figure S47 HMBC spectrum of **7** 31
Figure S48 ROESY spectrum of **7** 31
Figure S49 HRESIMS report of **7** 32
Figure S46 ^{1}H NMR spectrum of **13** (500 MHz, $CDCl_3$) 33
Figure S47 ^{13}C NMR spectrum of **13** (125 MHz, $CDCl_3$) 33
Figure S48 ^{1}H NMR spectrum of **17** (500 MHz, $CDCl_3$) 34
Figure S49 ^{13}C NMR spectrum of **17** (125 MHz, $CDCl_3$) 34
Figure S50 HSQC spectrum of **17** 35
Figure S51 ^{1}H NMR spectrum of **20** (500 MHz, $CDCl_3$) 35
Figure S52 ^{13}C NMR spectrum of **20** (125 MHz, $CDCl_3$) 36
Figure S53 HSQC spectrum of **20** 36
2. Computational details for **1** and **2** 37
3. Cytokine Analysis by ELISA of Induced T cells 38
4. EdU results for **7** and **21** 38
References: 39

1. Spectroscopic data

Table S1 ^{1}H (500 MHz) and ^{13}C NMR (125 MHz) data of compounds 13, 17 and 20 ($CDCl_3$, δ in ppm, J in Hz)

no.	**13**		**17**		**20**	
	δ_C	δ_H	δ_C	δ_H	δ_C	δ_H
1	42.6	1.99, dd (13.6, 7.2)<br>1.32, dd (13.6, 11.1)	46.6	3.10, dd (15.1, 7.9)<br>1.41, dd (15.1, 10.3)	49.2	2.34, overlapped;<br>1.70, dd (16.3, 14.0)
2	36.7	2.49, m	38.6	2.19, m	38	2.33, overlapped
3	83.3	4.92, dd (7.6, 6.9)	82.8	5.30, d (10.3)	80.9	5.60, dd (4.3, 3.7)
4	44.9	3.20, dd (9.6, 7.6)	43.1	3.26, dd (10.3, 7.6)	50.2	2.90, dd (8.6, 4.3)
5	121.2	5.54, d (9.6)	137.0	6.76, d (7.6)	140.4	7.00, d (8.6)
6	138.6	/	134.5	/	138	/
7	73.8	5.02, dd (9.1, 3.2)	195.7	/	199.5	/
8	41.4	2.89, dd (13.8, 9.1)<br>2.76, dd (13.8, 3.4)	50.2	4.50, d (15.1)<br>3.02, d (15.1)	36.3	3.28, dd (12.7, 1.8)<br>2.44, dd (12.7, 10.1)
9	207.7	/	205.2	/	77.5	4.87, dd (9.9, 1.8)
10	51.2	/	49.9	/	40.1	/
11	132.8	5.33, d (15.8)	137	5.51, d (15.9)	138.4	5.13, d (16.0)
12	132.9	5.56, dd (15.8, 8.8)	133.2	5.30, dd (15.9, 10.1)	128.8	5.67, dd (15.9, 8.9)
13	39.6	2.35, m	39.1	2.49, m	39.8	2.35, overlapped
14	79.2	4.99, d (8.7)	75.7	5.89, d (1.2)	81.5	5.01, d (2.9)
15	84.8	/	92.4	/	84.5	/
16	18.3	1.13, d (6.8)	19.2	0.91, d (7.0)	14.5	0.98, d (7.3)
17	17.8	1.71, d (1.1)	12.3	1.83, s	12.4	1.84, s
18	21.4	1.11, s	25.8	1.15, s	19.9	0.93, s
19	24.3	1.18, s	22.1	1.32, s	24.0	1.17, s
20	19.3	0.98, d (6.8)	21.1	1.18, d (7.1)	26.1	0.93, d (6.1)
1'	165.9	/	165.6	/	165.9	/
2'	130.4	/	130.3	/	130.1	/
3',7'	129.5	7.91, dd (7.1, 1.3)	129.4	7.91, d (7.3)	129.9	8.04, d (7.3)
4',6'	128.4	7.38, t (7.8)	128.6	7.42, t (7.7)	128.7	7.45, t (7.7)
5'	133.1	7.52, t (7.4)	134.4	7.55, t (7.4)	133.2	7.56, t (7.5)
7-	169.7	/				
OAc	20.7	1.63, s				
9-					170.5	/
OAc					21.2	2.06, s
14-	171.0	/	170.1		170.7	/
OAc	21.2	2.17, s	21.6	2.23, s	21.0	2.23, s
15-			170.1			
OAc			25.2	2.15, s		

Figure S1 1H NMR spectrum of 1 (500 MHz, $CDCl_3$)

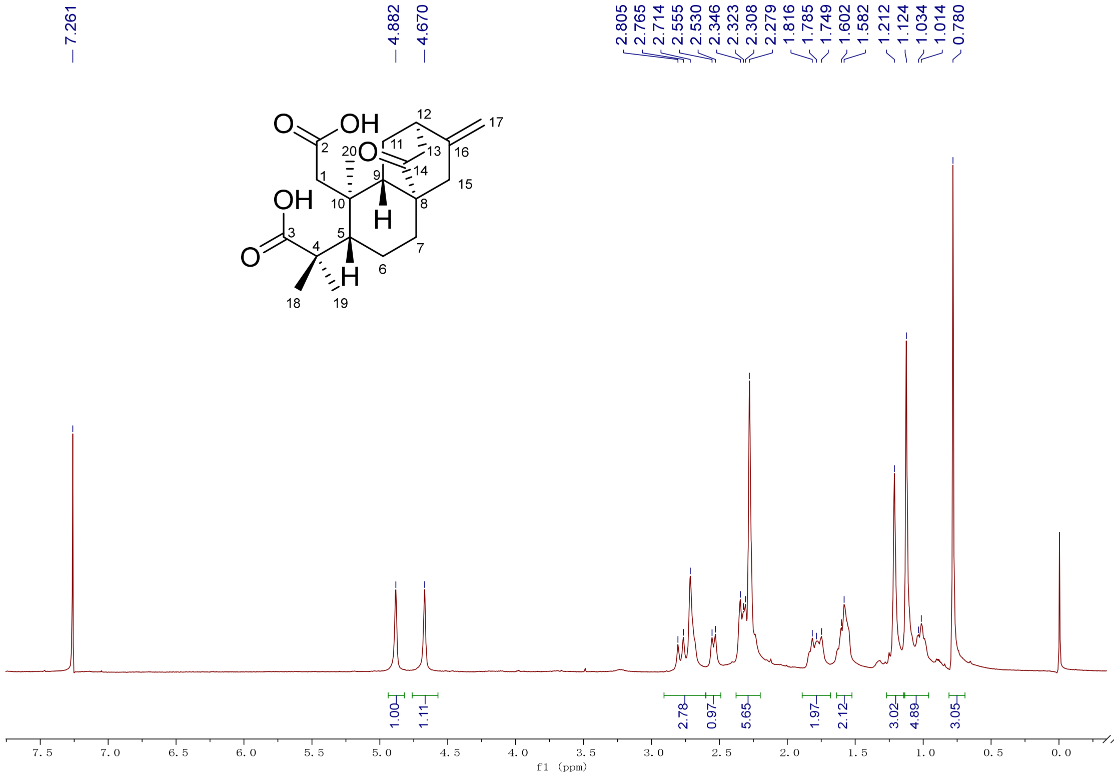

Figure S2 ^{13}C and DEPT 135 NMR spectra of 1 (125 MHz, $CDCl_3$)

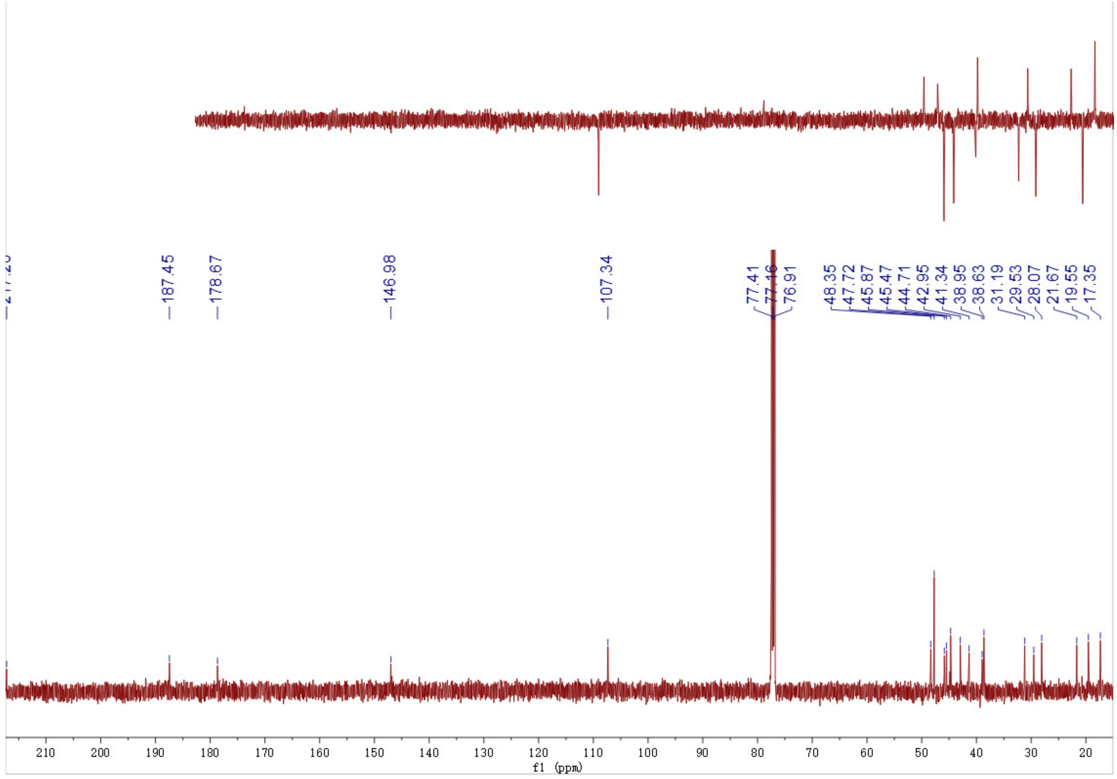

Figure S3 HSQC spectrum of 1

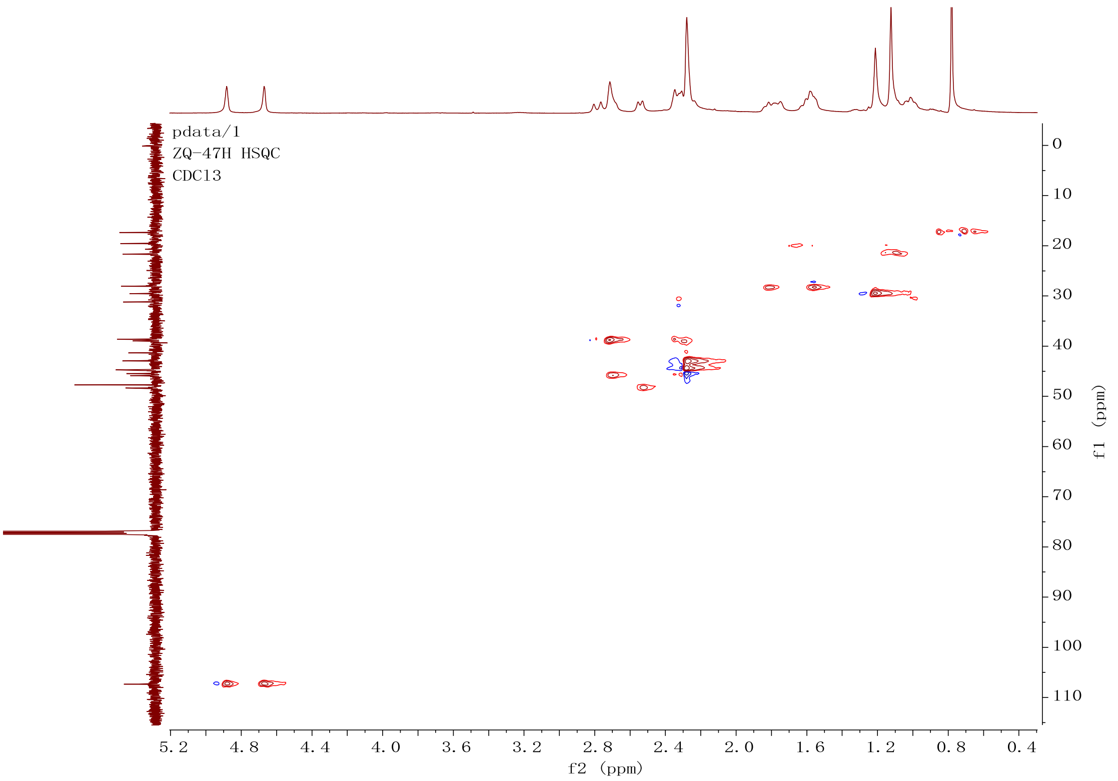

Figure S4 ^{1}H-^{1}H COSY spectrum of 1

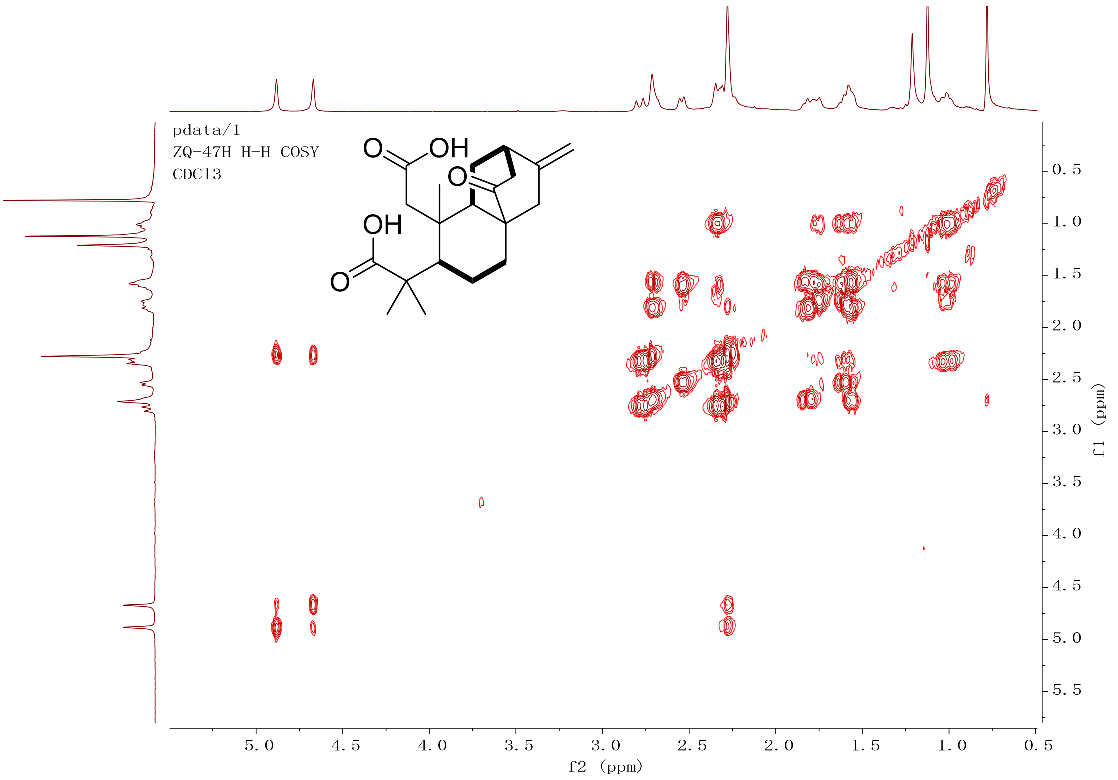

Figure S5. HMBC spectrum of 1

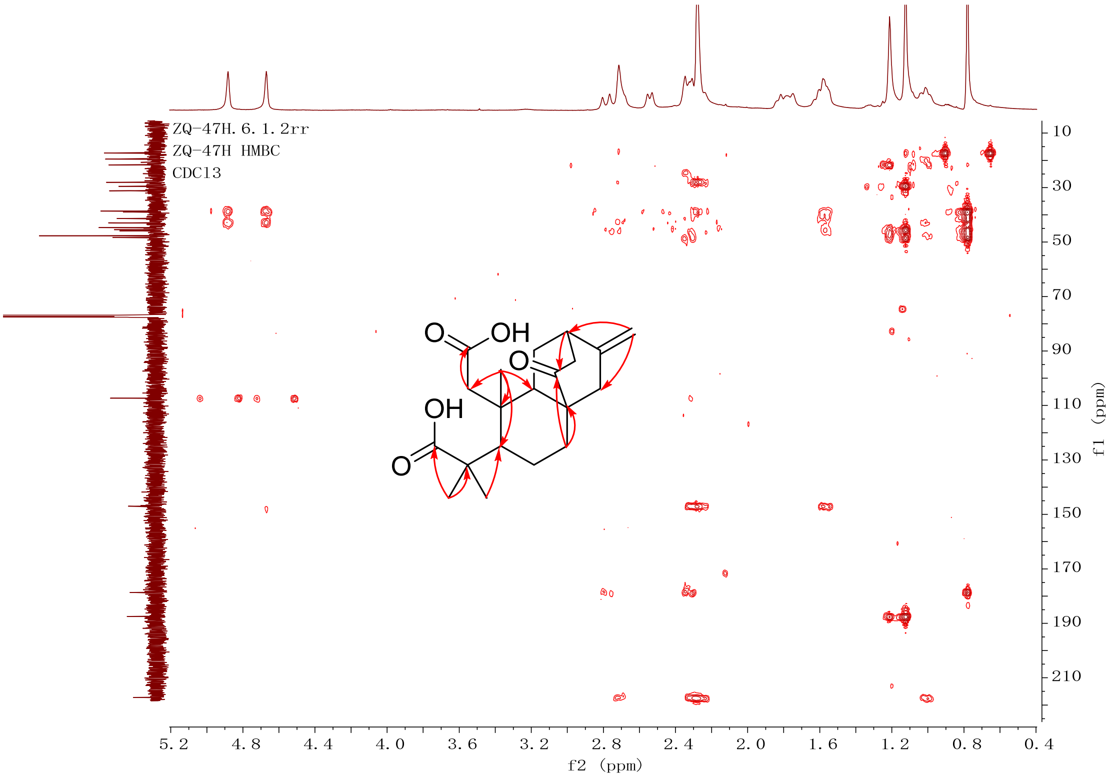

Figure S6 ROESY spectrum of 1

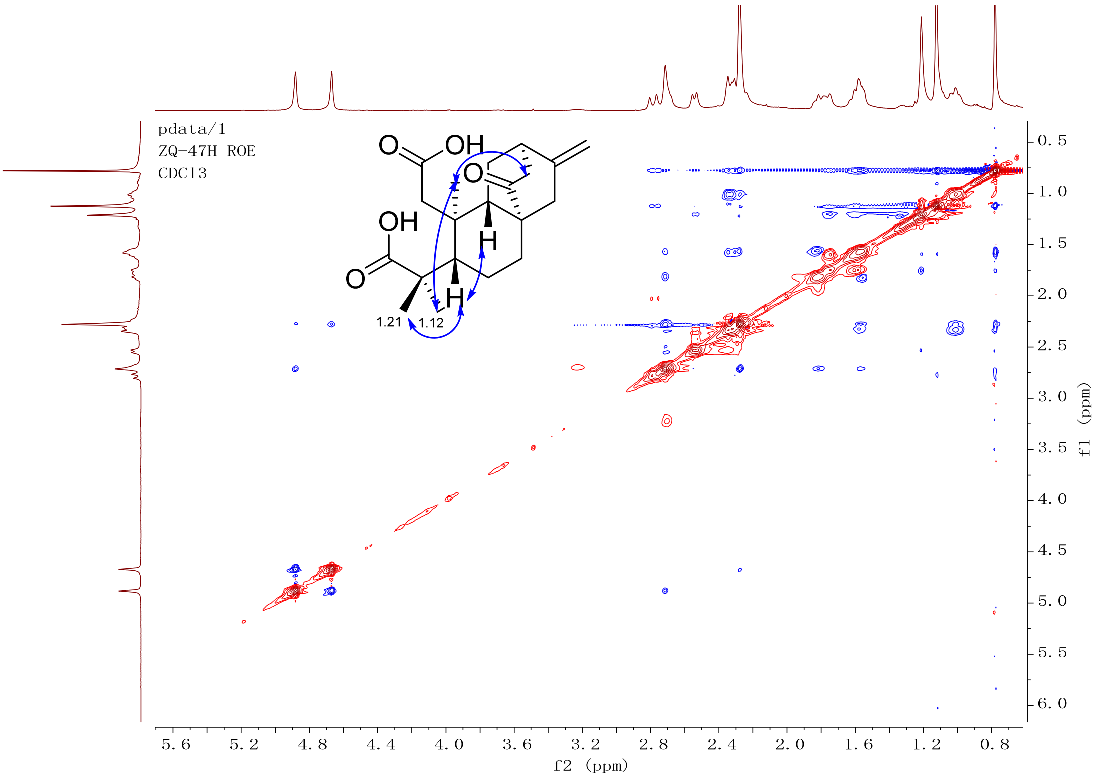

Figure S7 HRESIMS report of 1

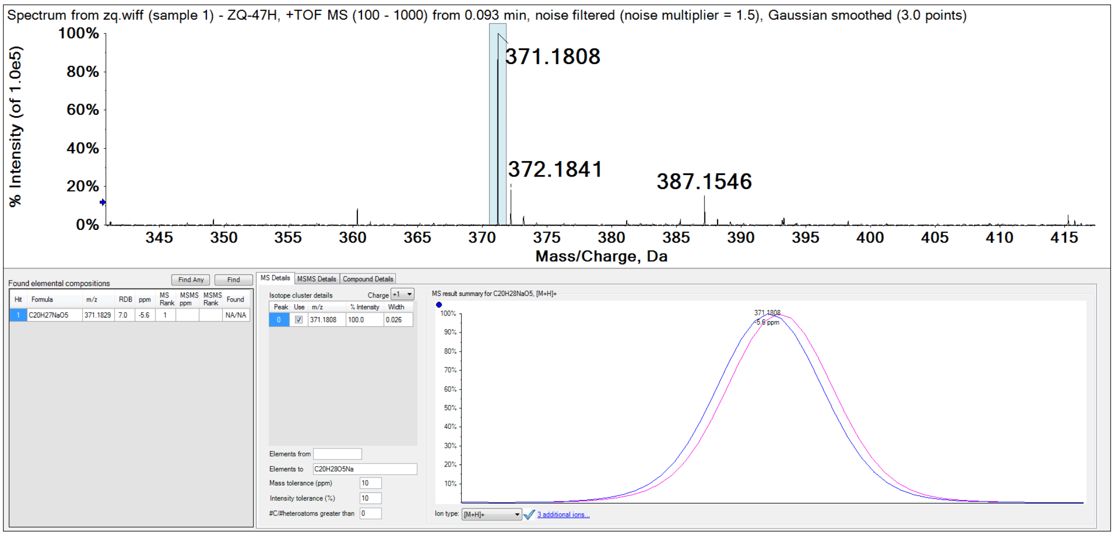

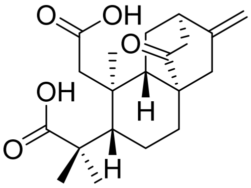

Chemical Formula: $C_{20}H_{28}O_5$

Exact Mass: 348.19

Figure S8 ^{1}H NMR spectrum of 2 (500 MHz,)

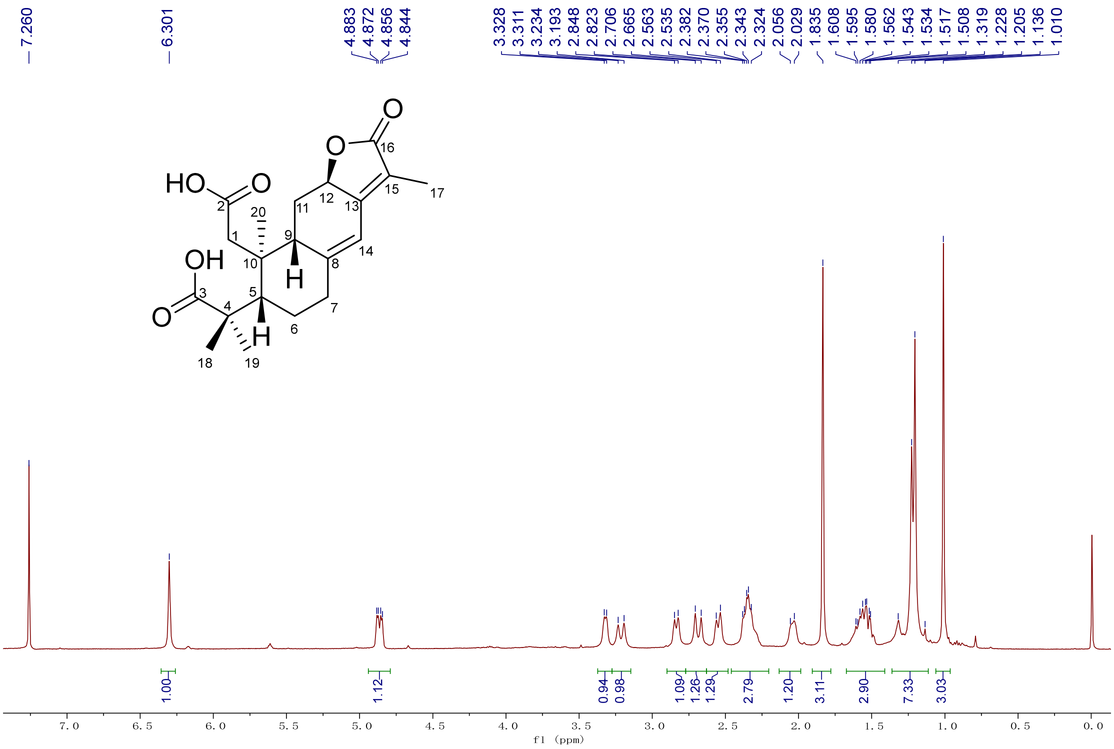

Figure S9 ^{13}C and DEPT 135 NMR spectra of 2 (125 MHz, $CDCl_3$)

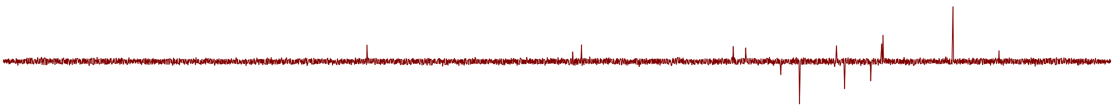

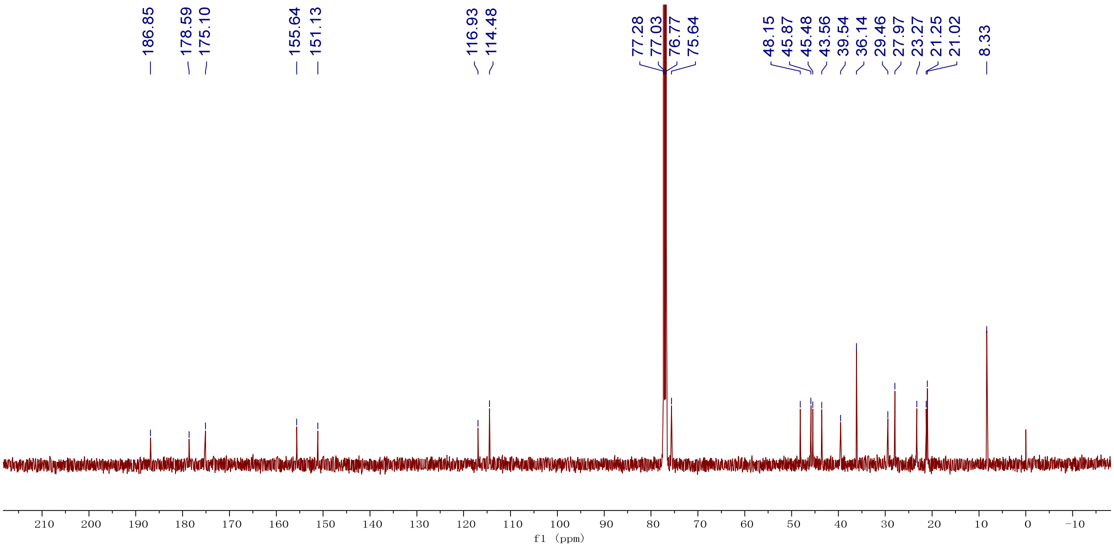

Figure S10 HSQC spectrum of 2

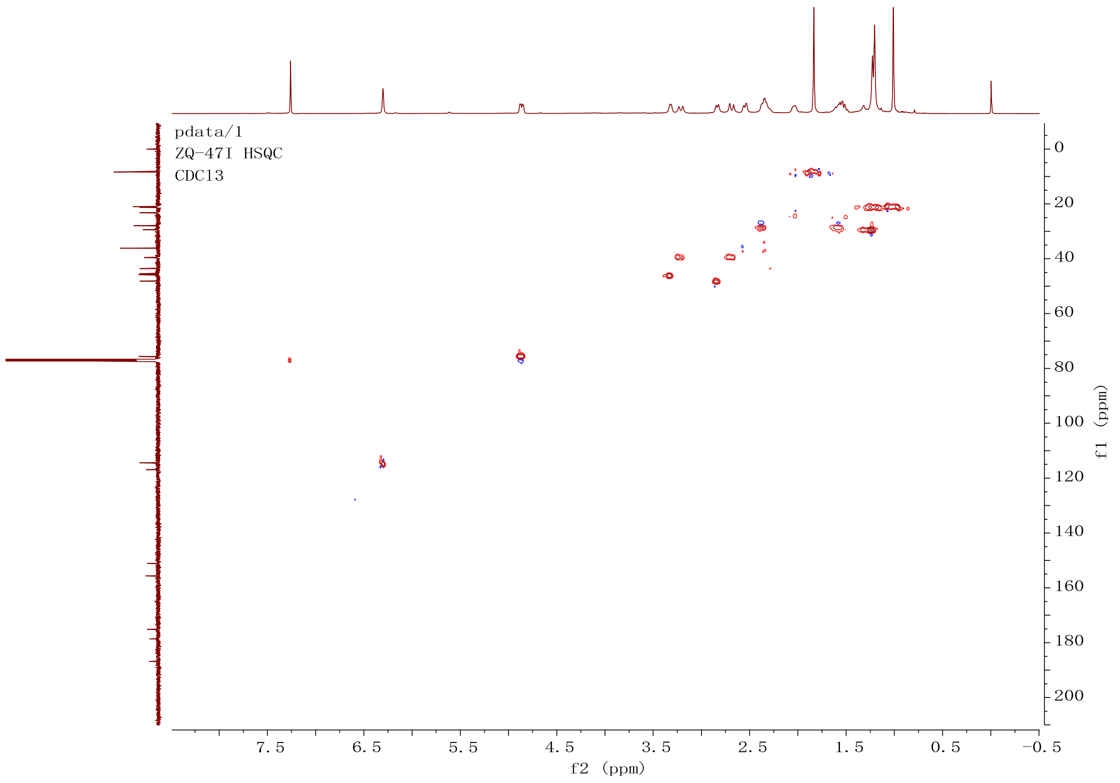

Figure S11 ^{1}H-^{1}H COSY spectrum of 2

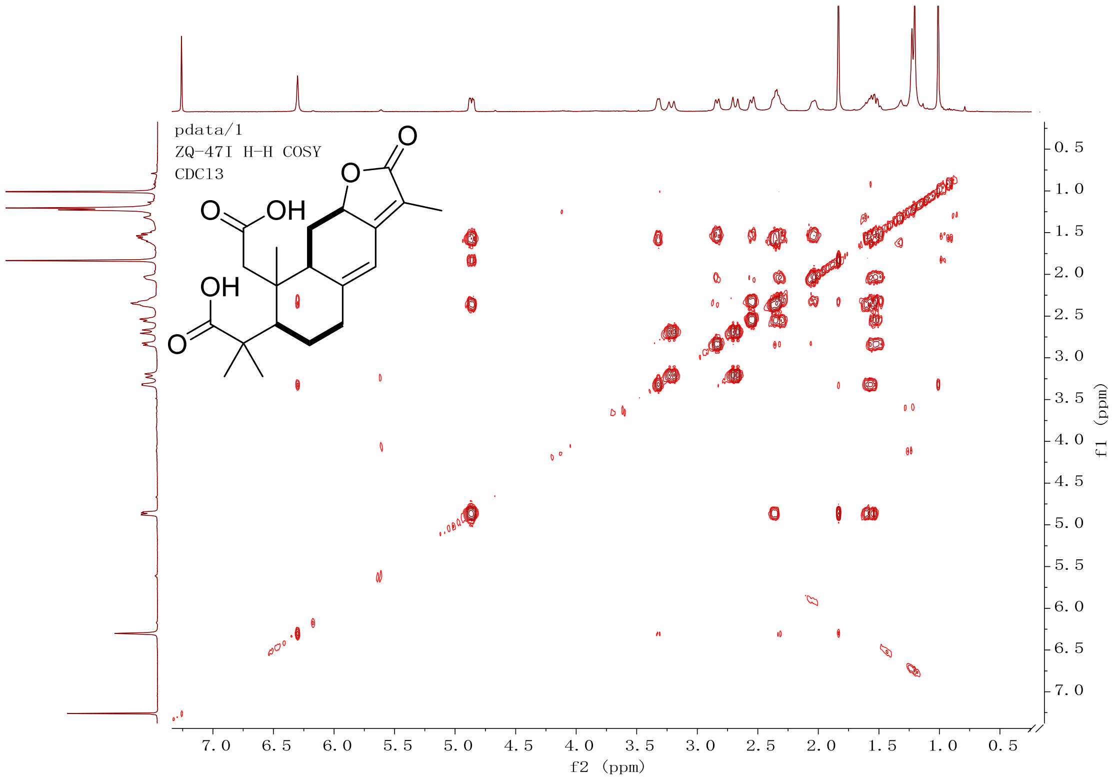

Figure S12 HMBC spectrum of 2

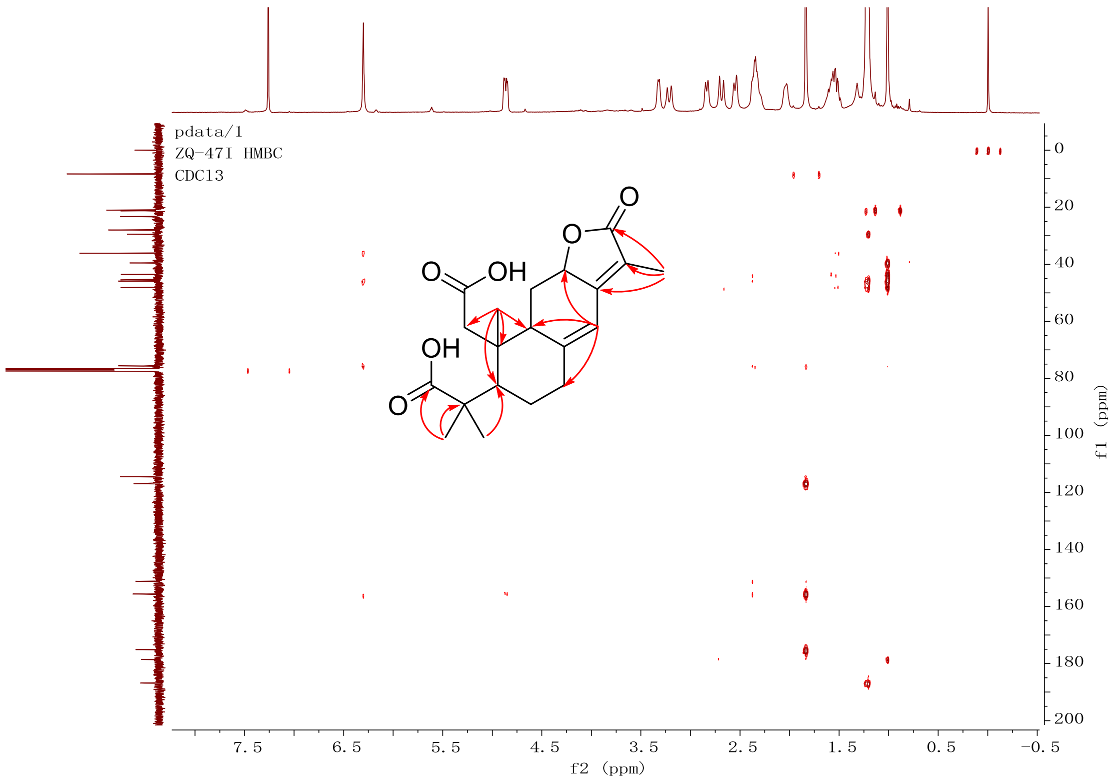

Figure S13 ROESY spectrum of 2

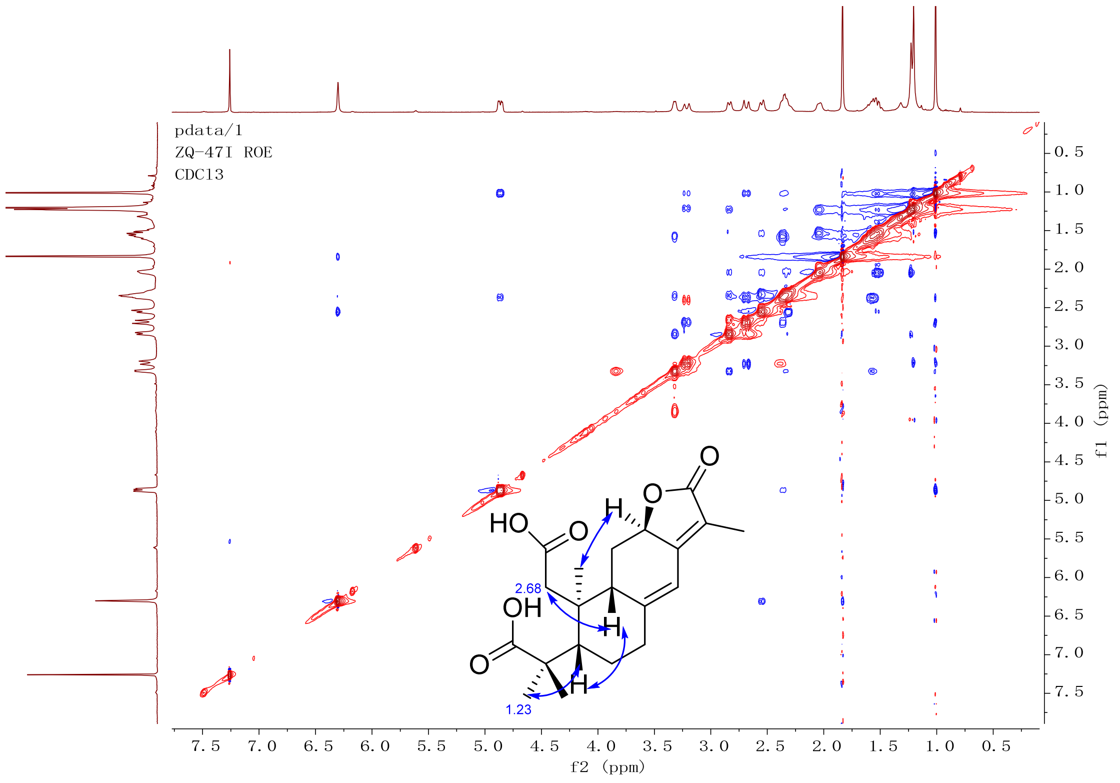

Figure S14 HRESIMS report of 2

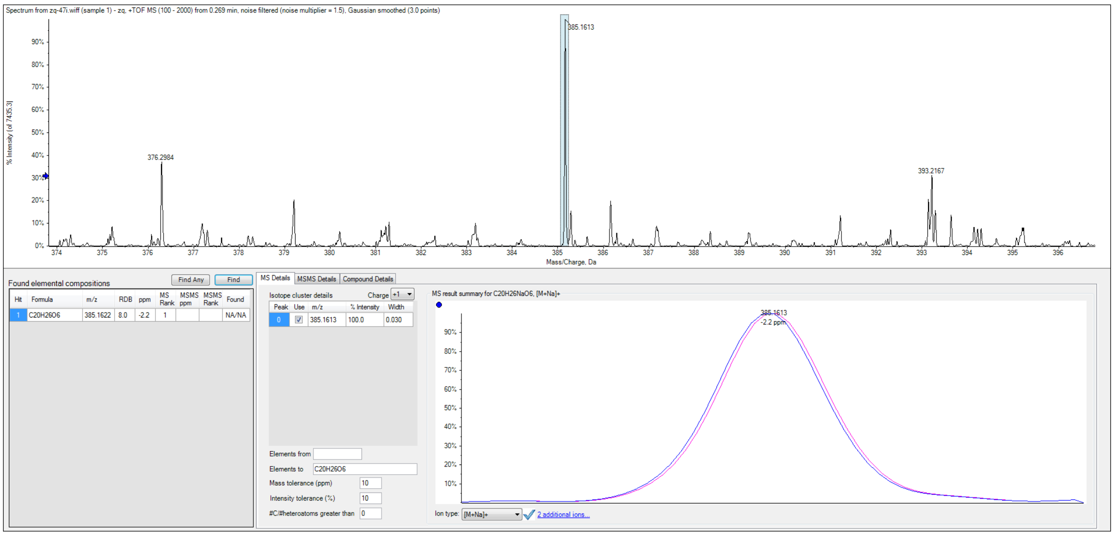

Hit	Formula	m/z	RDB	ppm	MS Rank	MSMS ppm	MSMS Rank	Found
1	C20H26O6	385.1622	8.0	-2.2	1			NA/NA

Peak	Use	m/z	% Intensity	Width
0	✓	385.1613	100.0	0.030

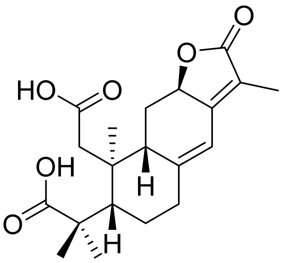

Chemical Formula: $C_{20}H_{26}O_6$
Exact Mass: 362.17

Figure S15 ^{1}H NMR spectrum of 3 (500 MHz, CDCl₃)

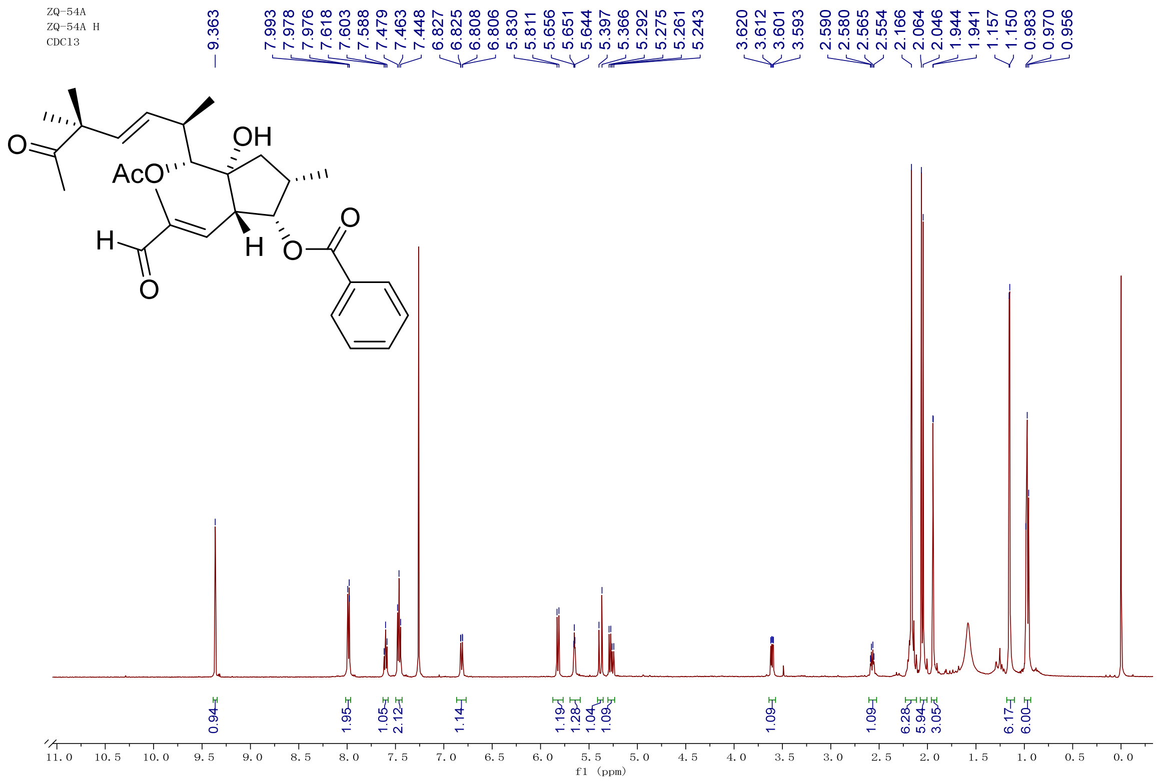

Figure S16 ^{13}C NMR spectrum of 3 (125 MHz, CDCl₃)

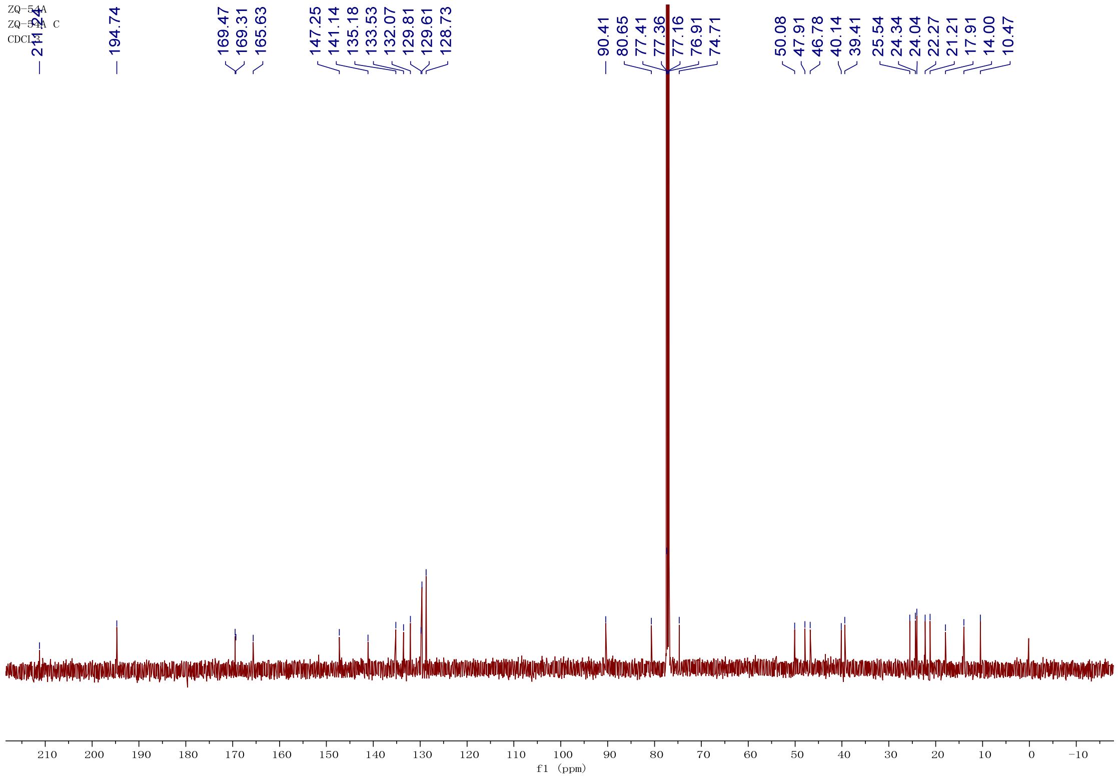

Figure S17 HSQC spectrum of 3

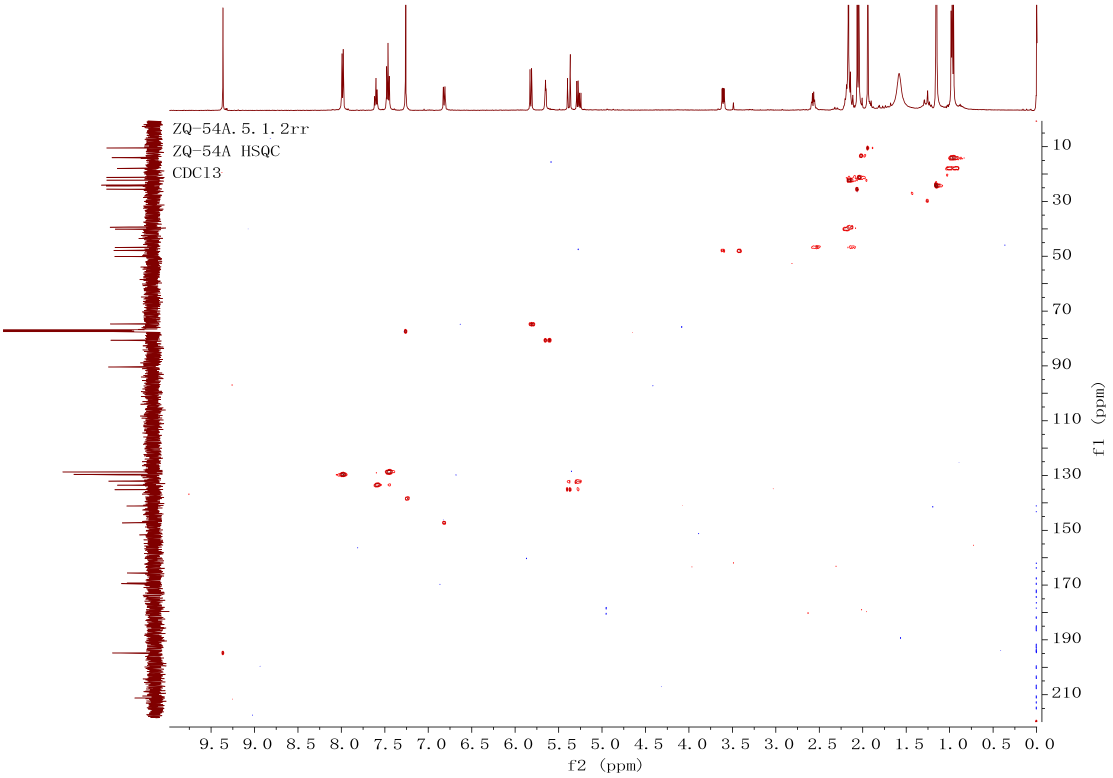

Figure S18 ^{1}H-^{1}H COSY spectrum of 3

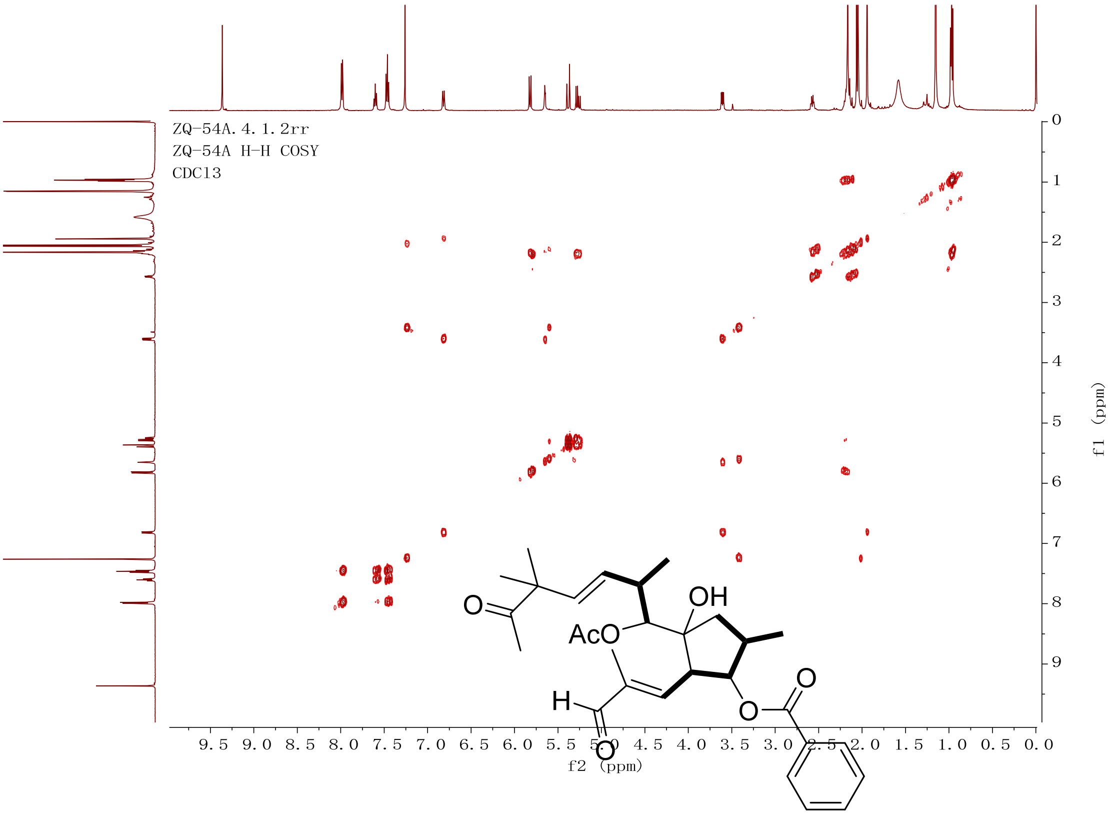

Figure S19 HMBC spectrum of 3

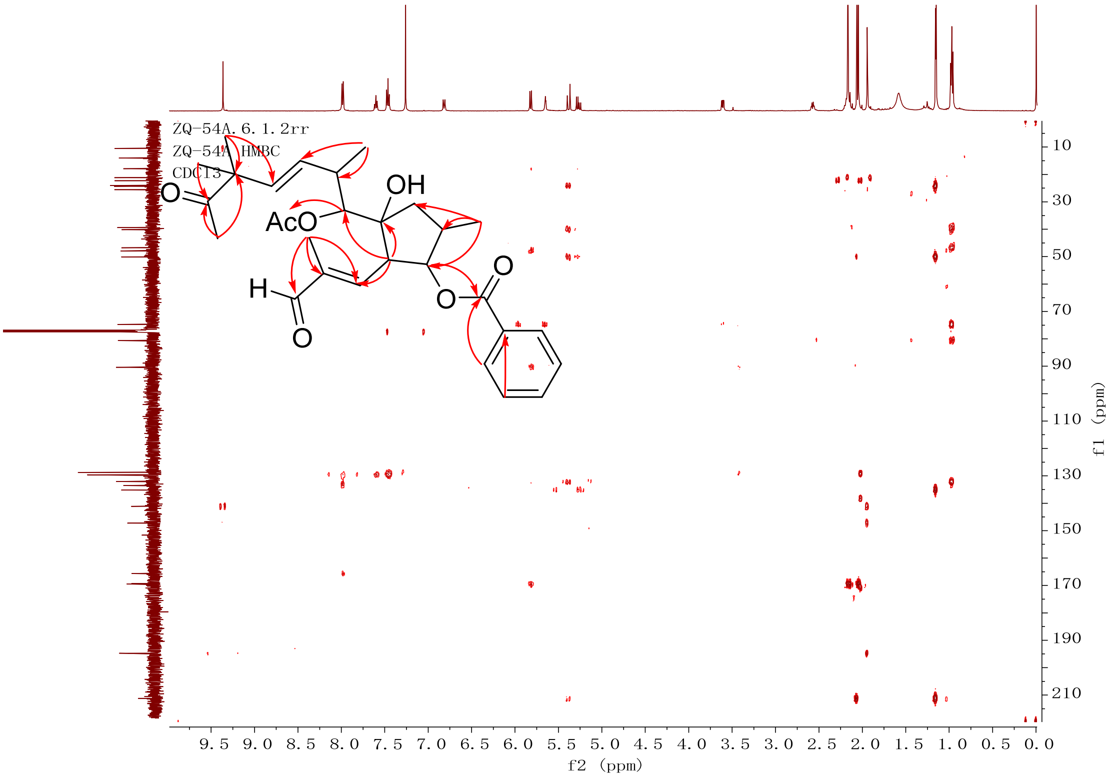

Figure S20 ROESY spectrum of 3

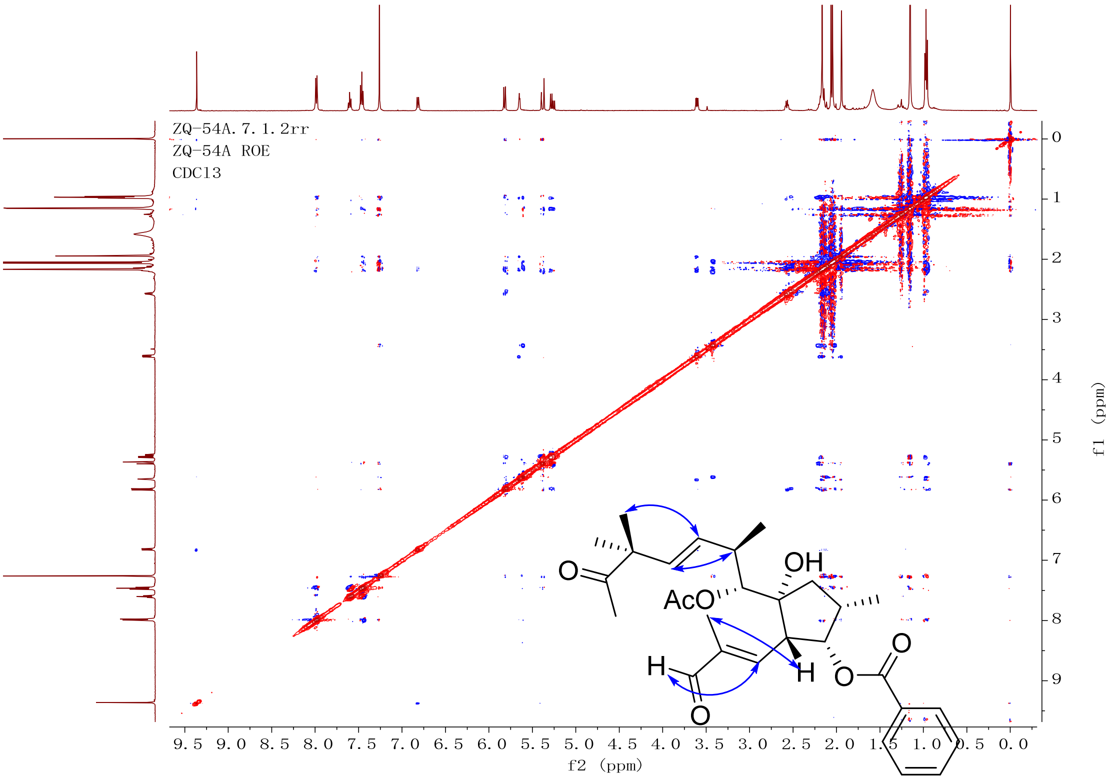

Figure S21 HRESIMS report of 3

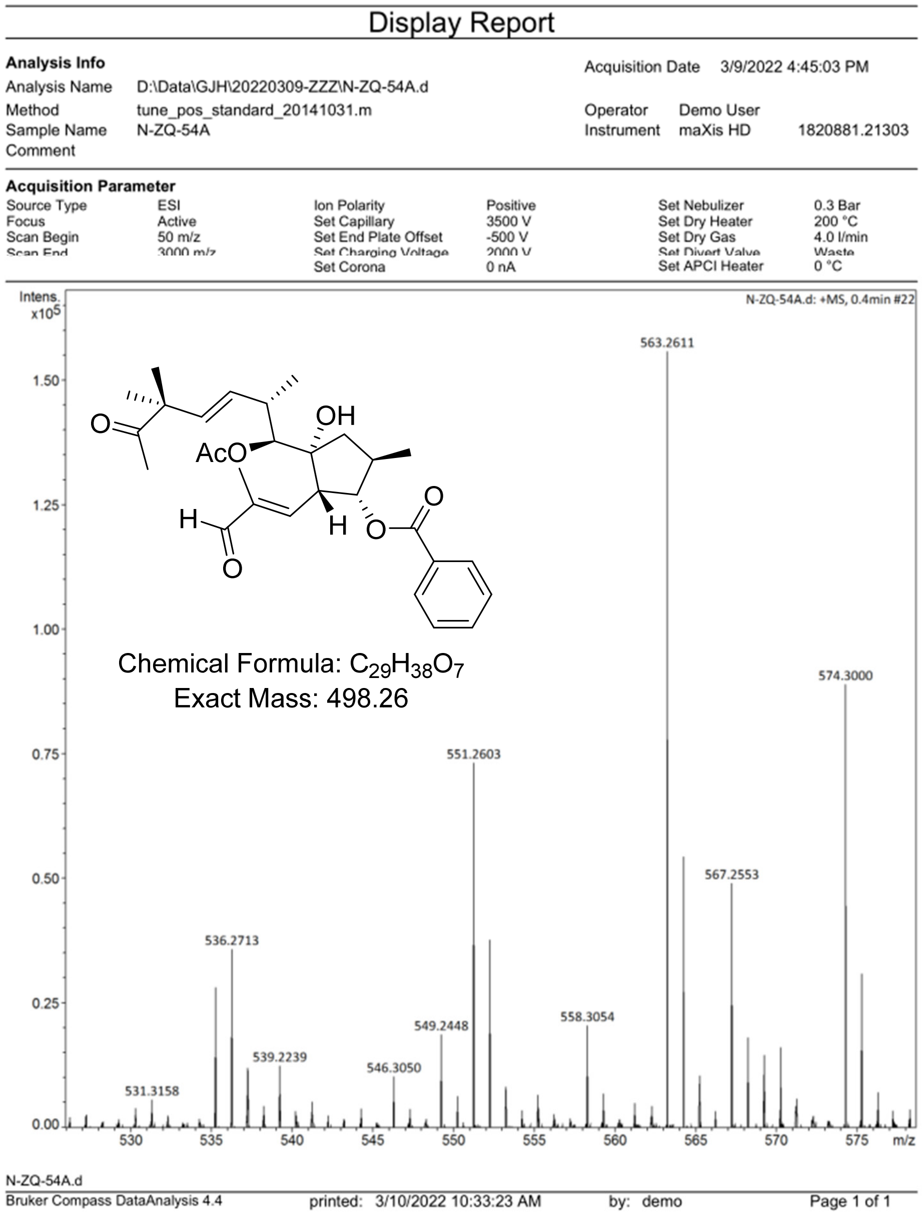

Figure S22 ^{1}H NMR spectrum of 4 (500 MHz, $CDCl_3$)

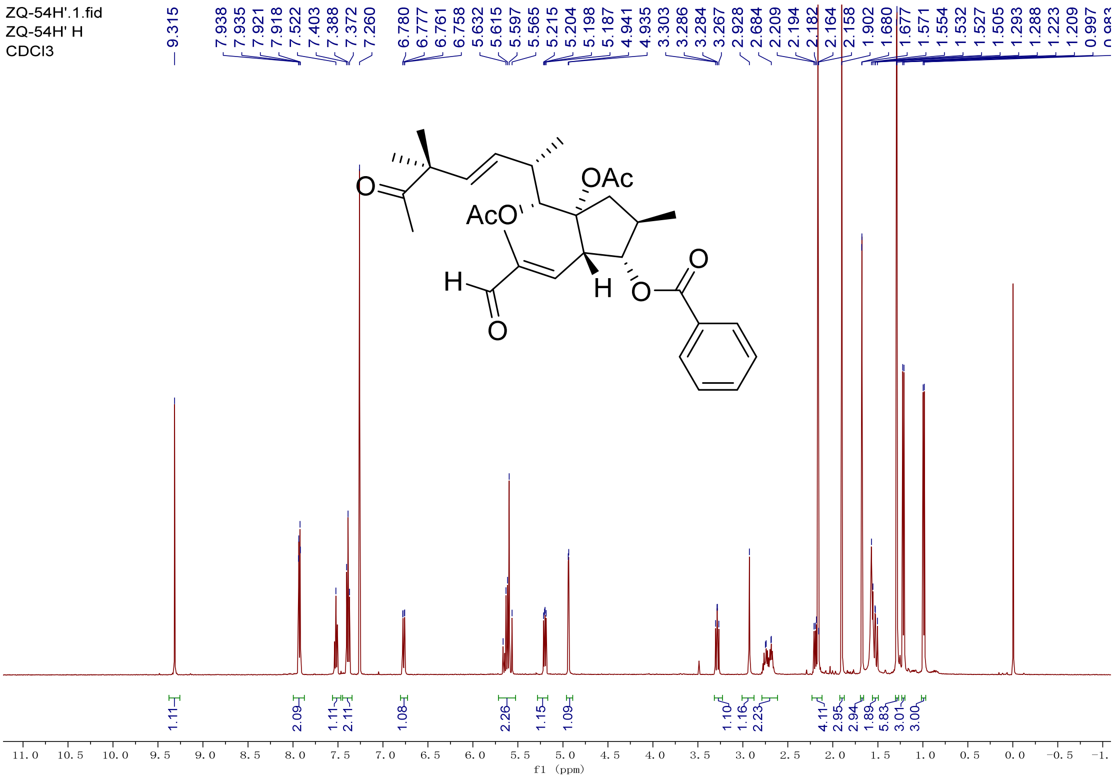

Figure S23 ^{13}C NMR spectra of 4 (125 MHz, $CDCl_3$)

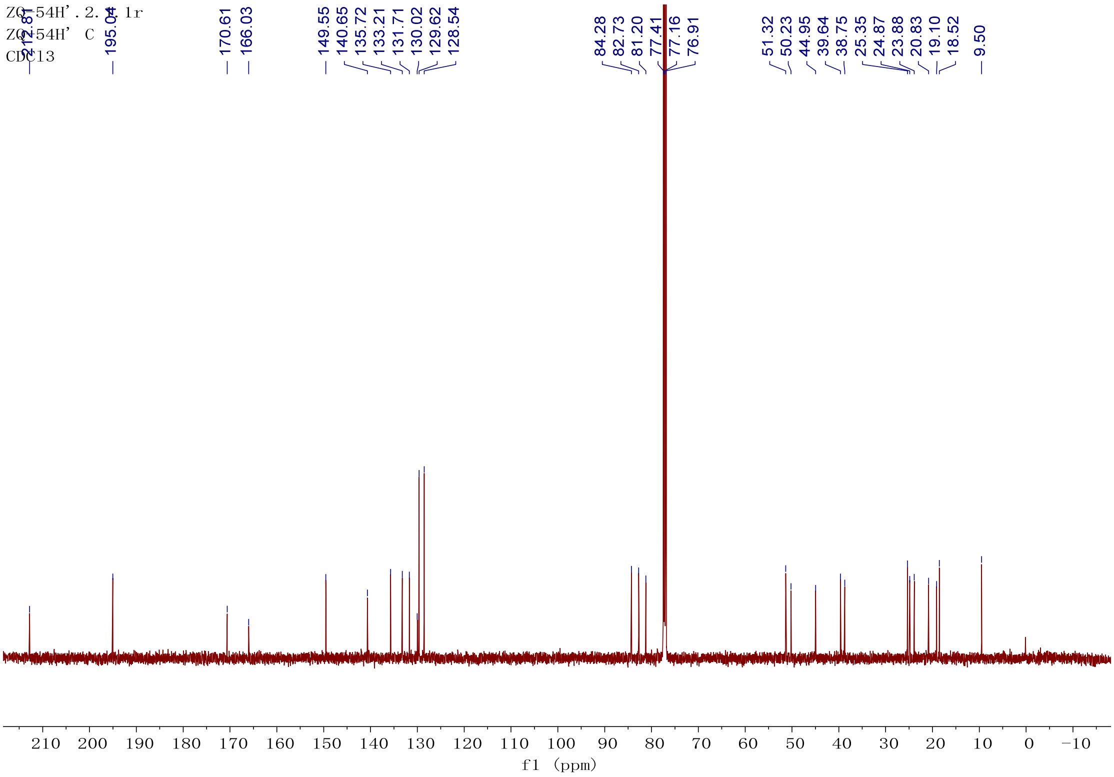

Figure S24. HSQC spectrum of 4

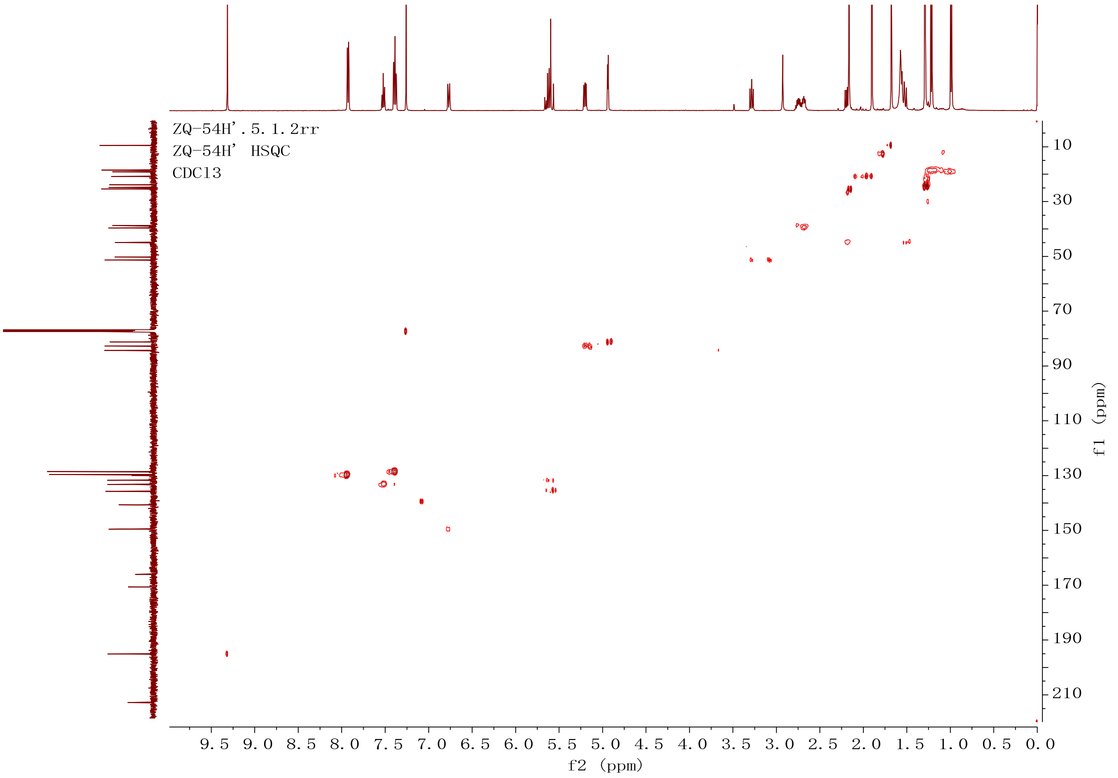

Figure S25 ¹H-¹H COSY spectrum of 4

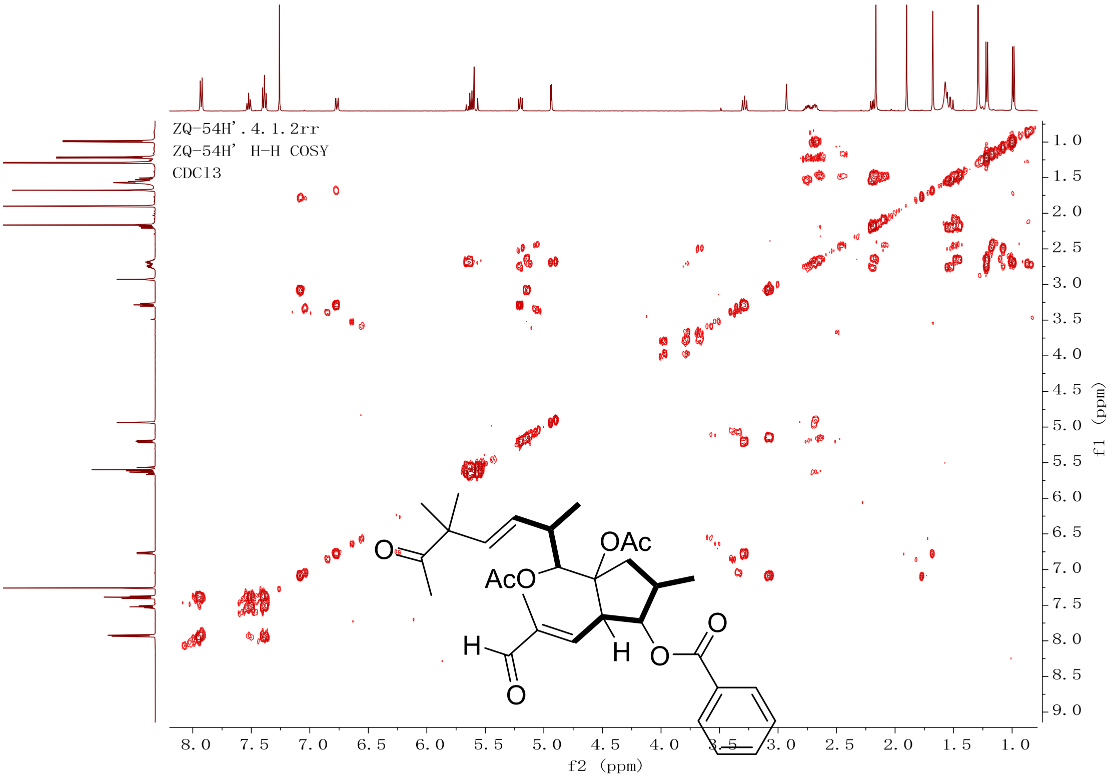

Figure S26 HMBC spectrum of 4

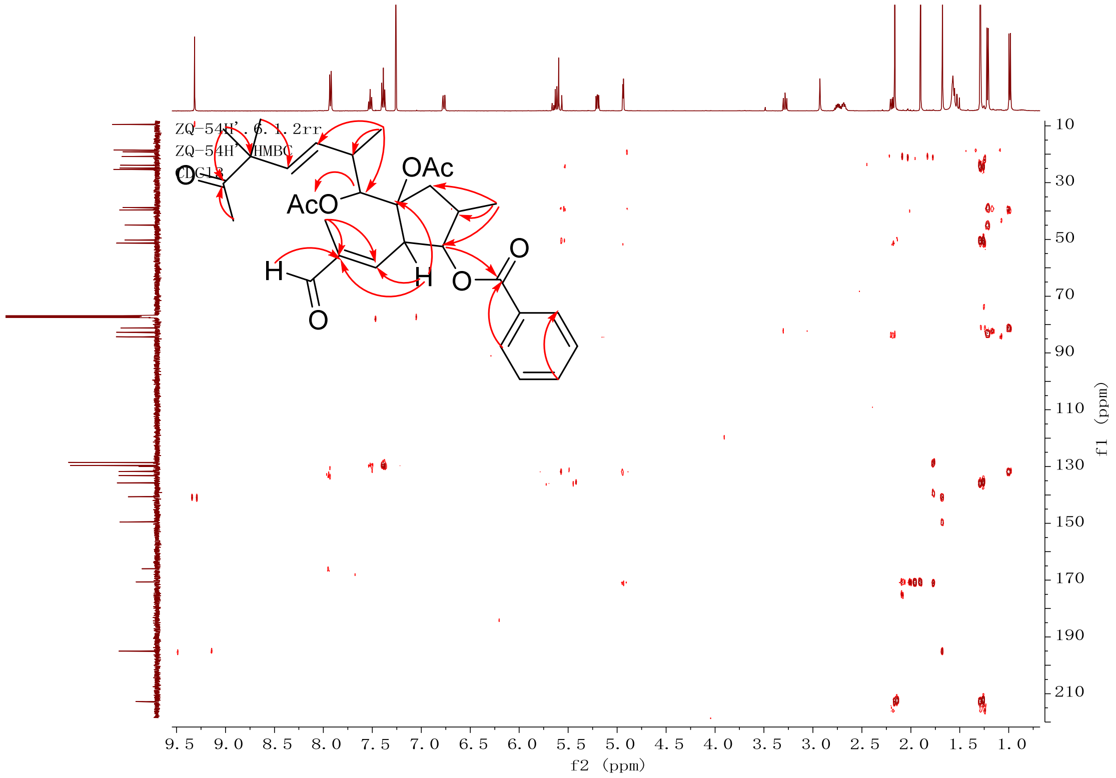

Figure S27 ROESY spectrum of 4

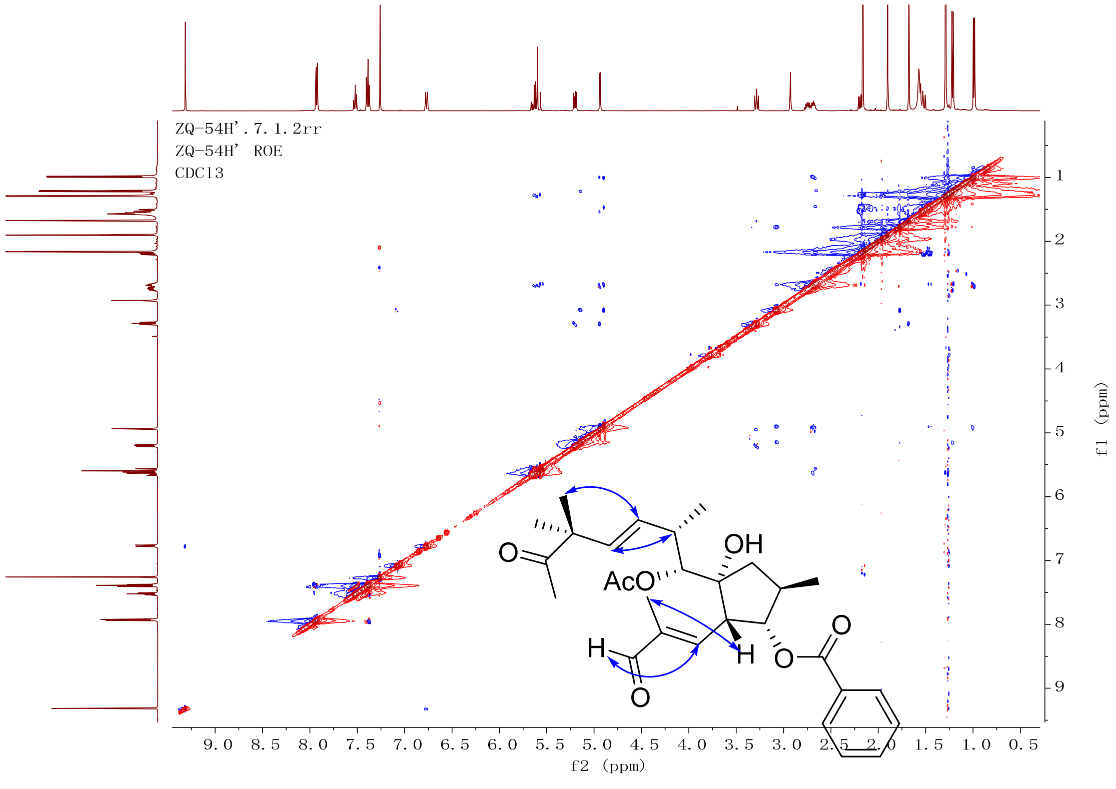

Figure S28 HRESIMS report of 4

Display Report

Analysis Info

Analysis Name	D:\Data\GJH\20220309-ZZZ\N-ZQ-54H1.d	Acquisition Date	3/9/2022 4:28:00 PM
Method	tune_pos_standard_20141031.m	Operator	Demo User
Sample Name	N-ZQ-54H1	Instrument	maXis HD 1820881.21303
Comment			

Acquisition Parameter

Source Type	ESI	Ion Polarity	Positive	Set Nebulizer	0.3 Bar
Focus	Active	Set Capillary	3500 V	Set Dry Heater	200 °C
Scan Begin	50 m/z	Set End Plate Offset	-500 V	Set Dry Gas	4.0 l/min
Scan End	3000 m/z	Set Charging Voltage	2000 V	Set Divert Valve	Waste
		Set Corona	0 nA	Set APCI Heater	0 °C

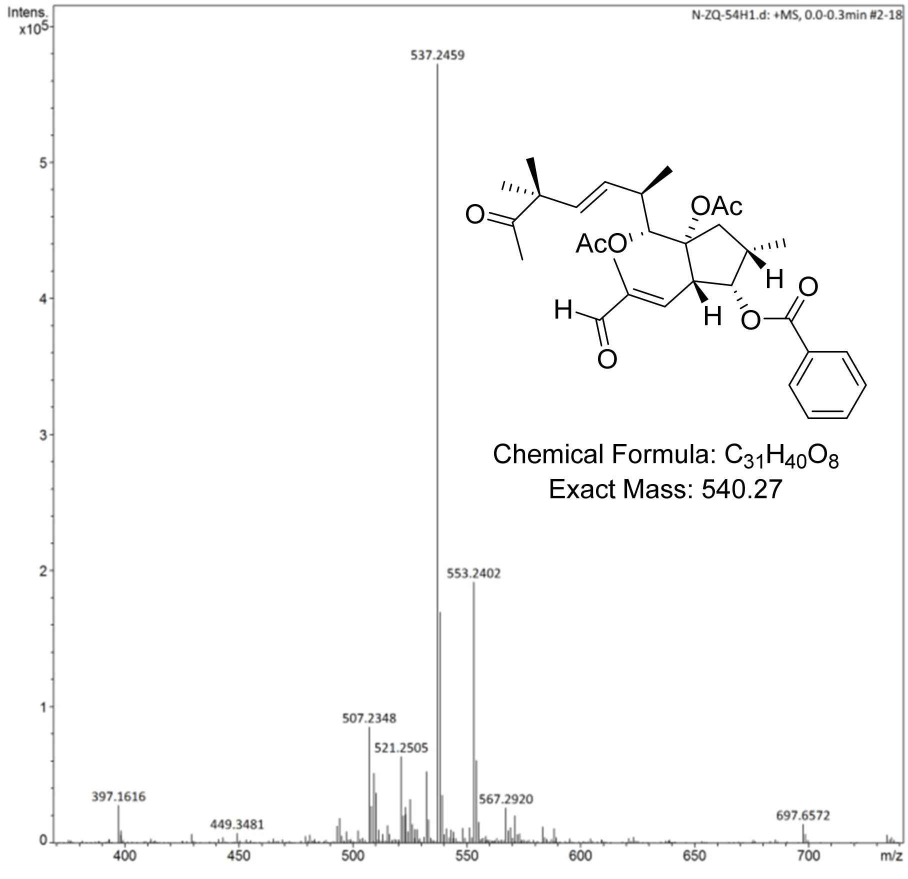

N-ZQ-54H1.d

Bruker Compass DataAnalysis 4.4 printed: 3/10/2022 9:57:43 AM by: demo Page 1 of 1

Figure S29 ^{1}H NMR spectrum of 5 (500 MHz, CDCl$_3$)

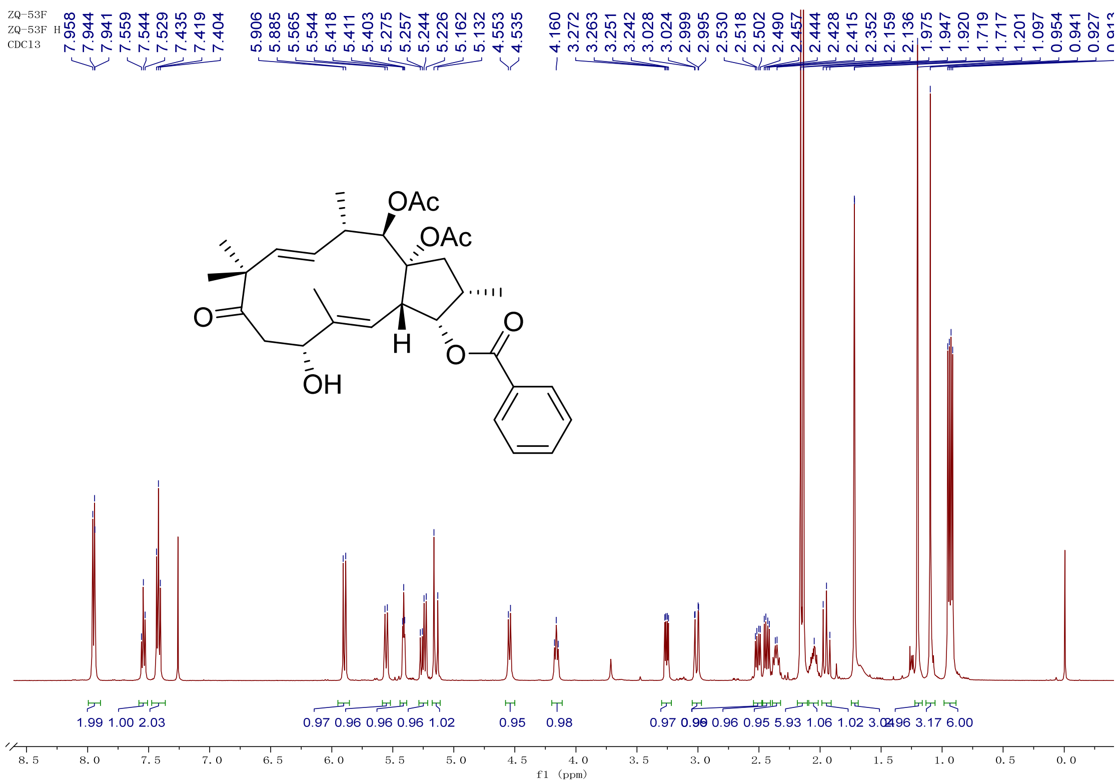

Figure S30 ^{13}C spectrum of 5 (125 MHz, CDCl$_3$)

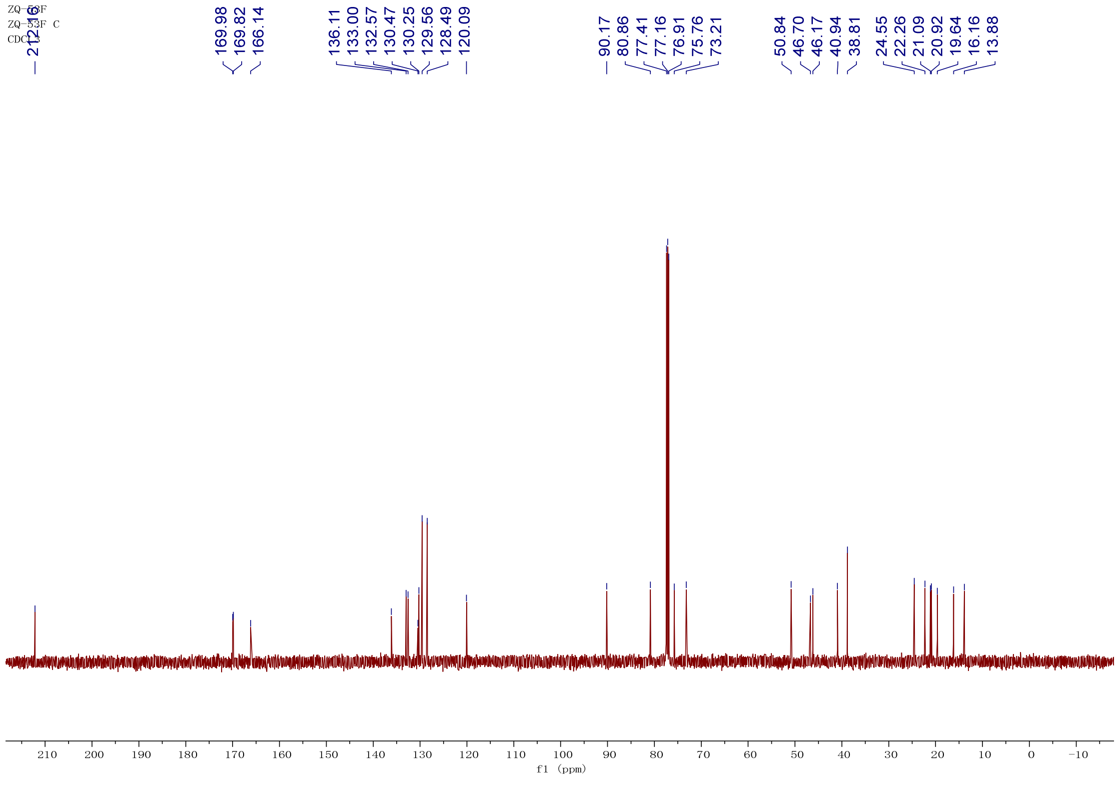

Figure S31 HSQC spectrum of 5

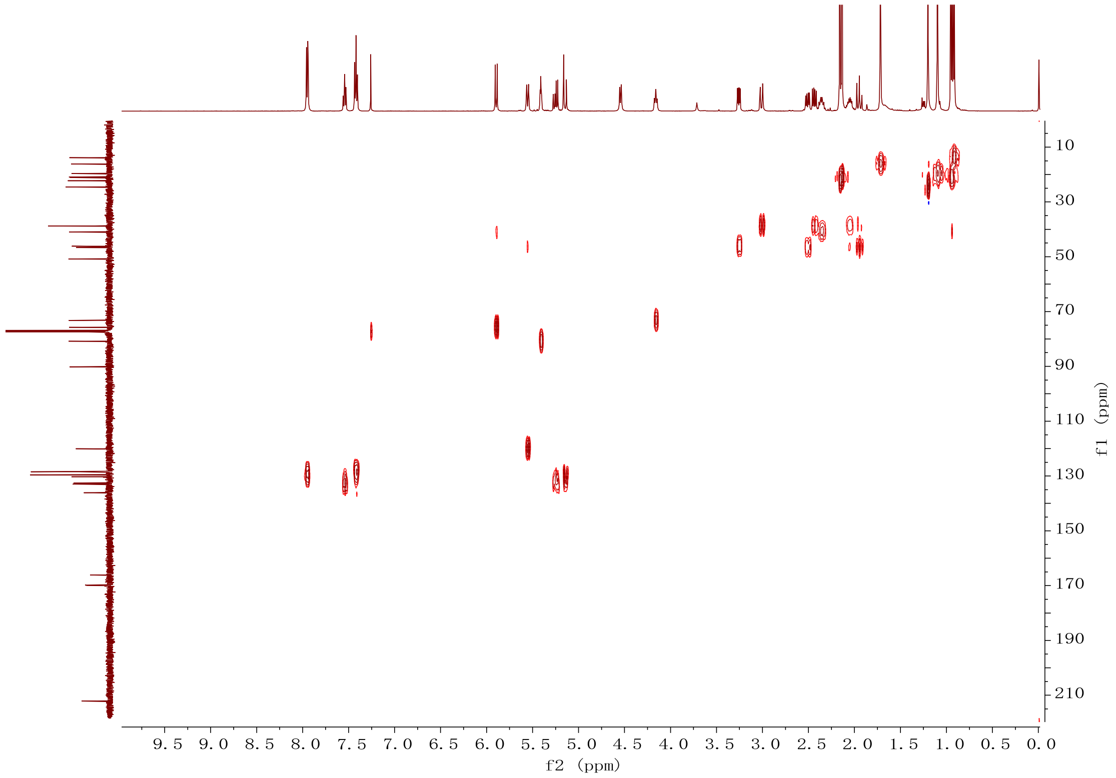

Figure S32 ^1H-^1H COSY spectrum of 5

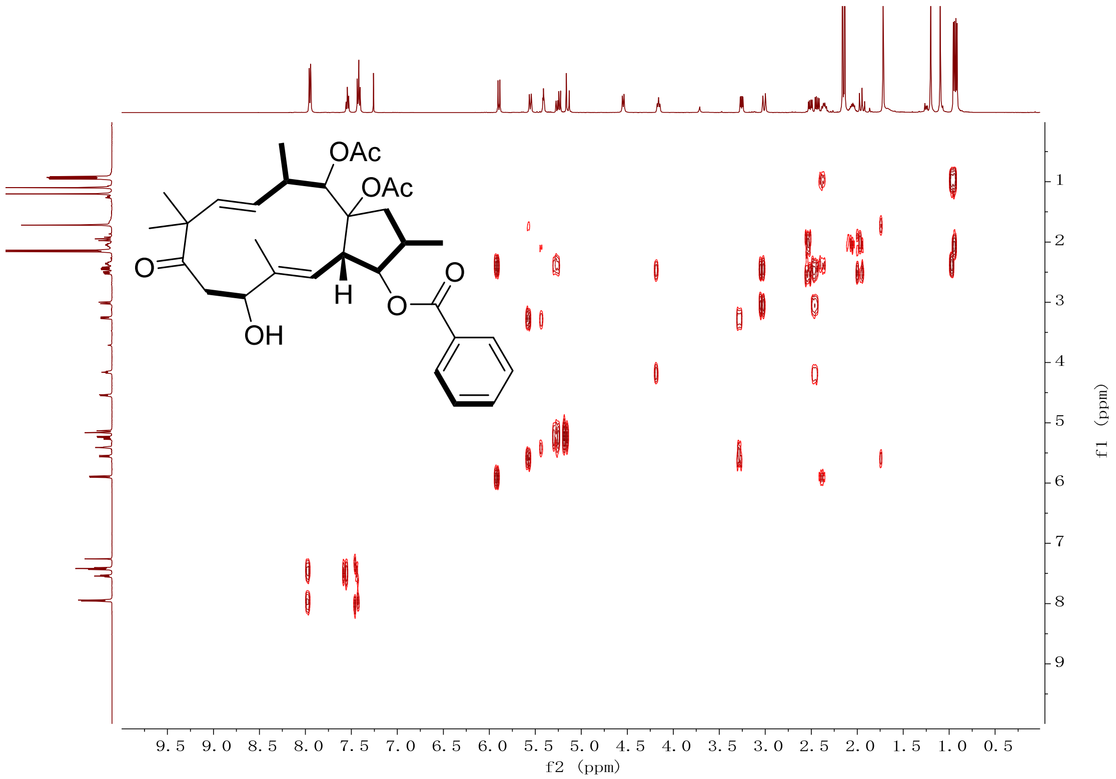

Figure S33 HMBC spectrum of 5

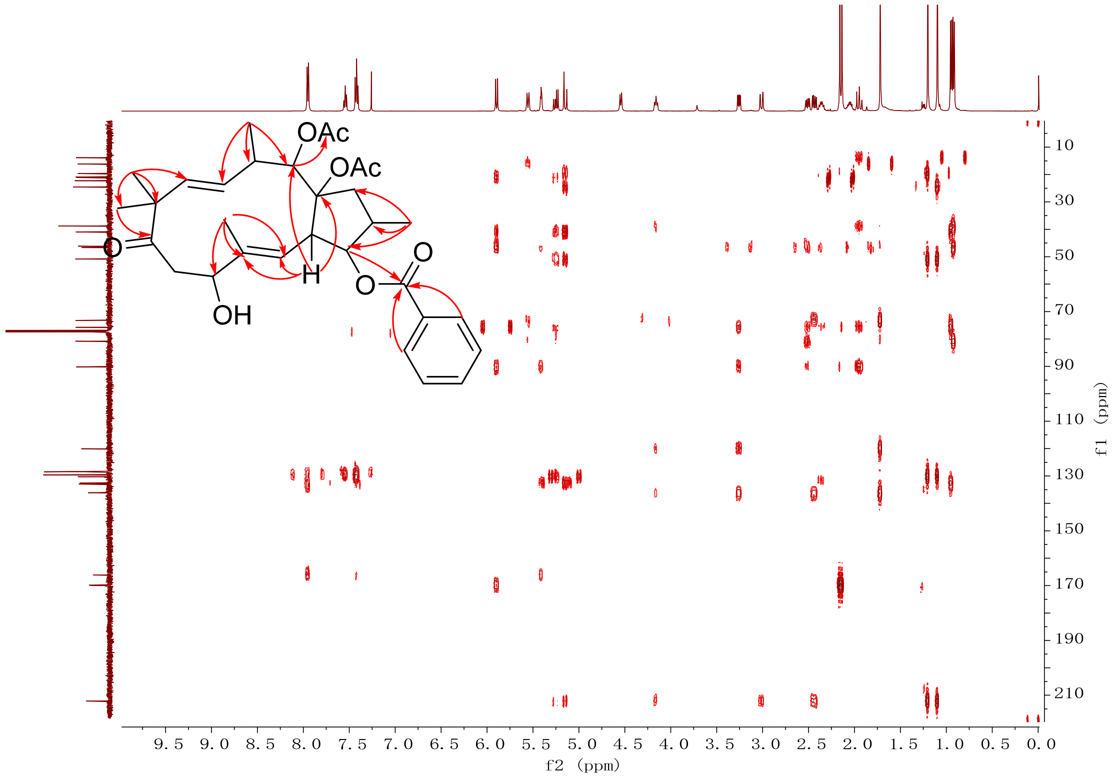

Figure S34 ROESY spectrum of 5

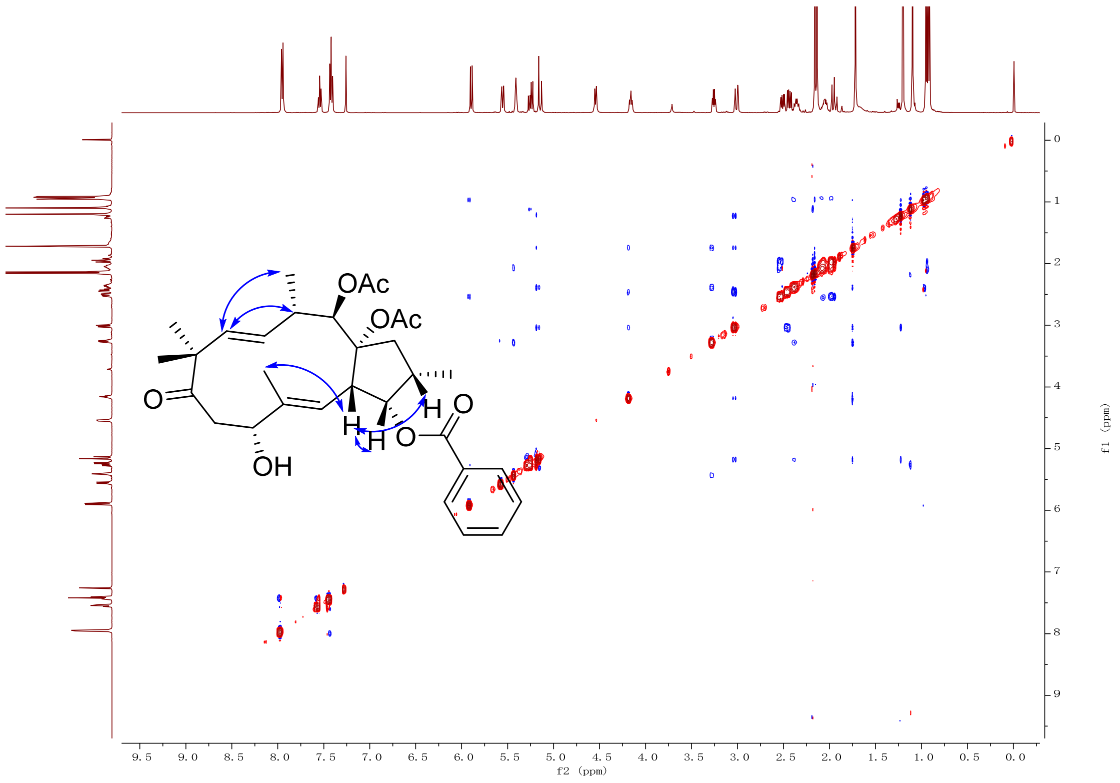

Figure S35 HRESIMS report of 5

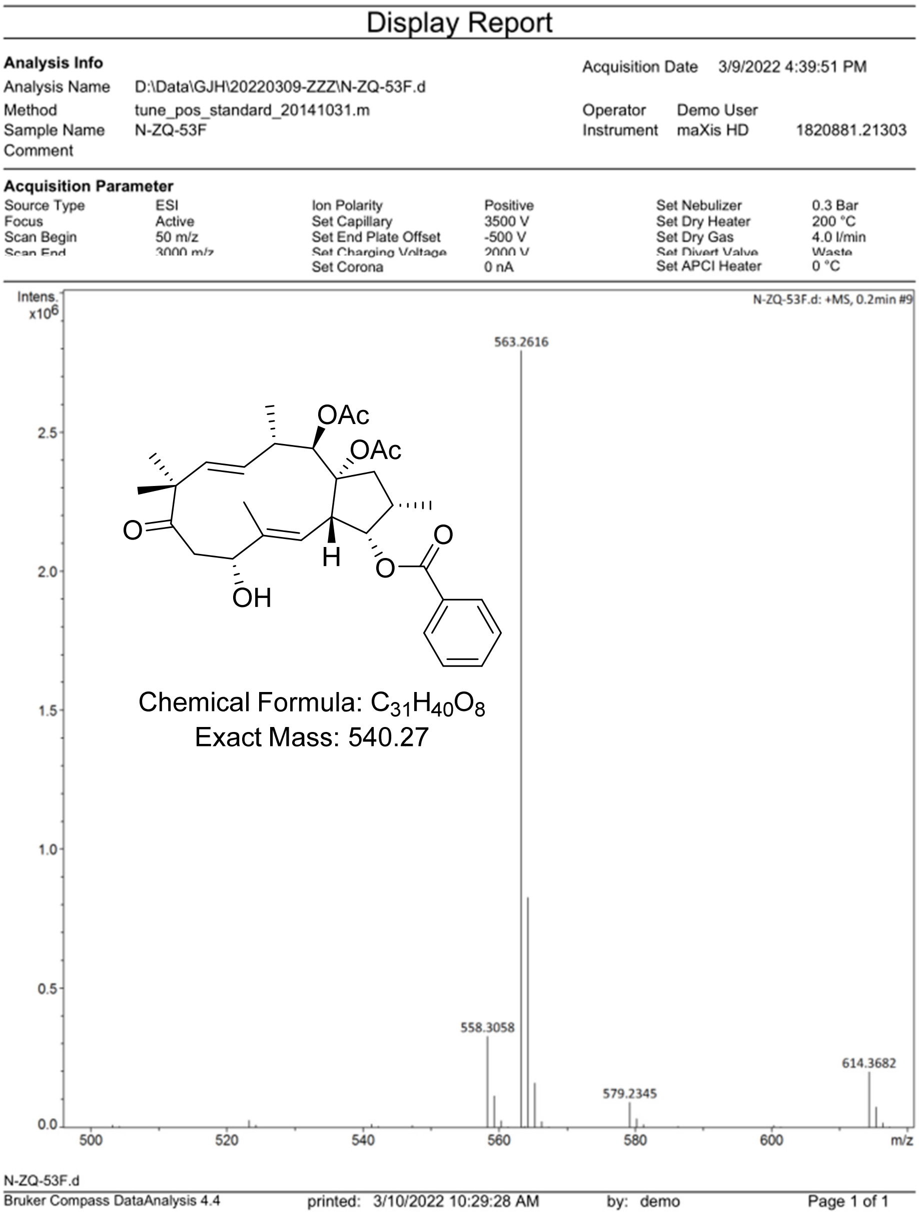

Figure S36 ^{1}H NMR spectrum of 6 (500 MHz, $CDCl_3$)

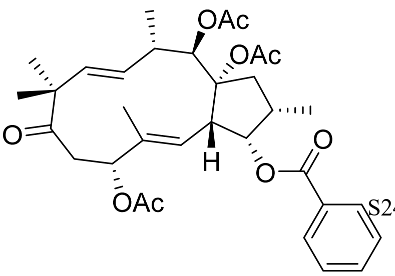

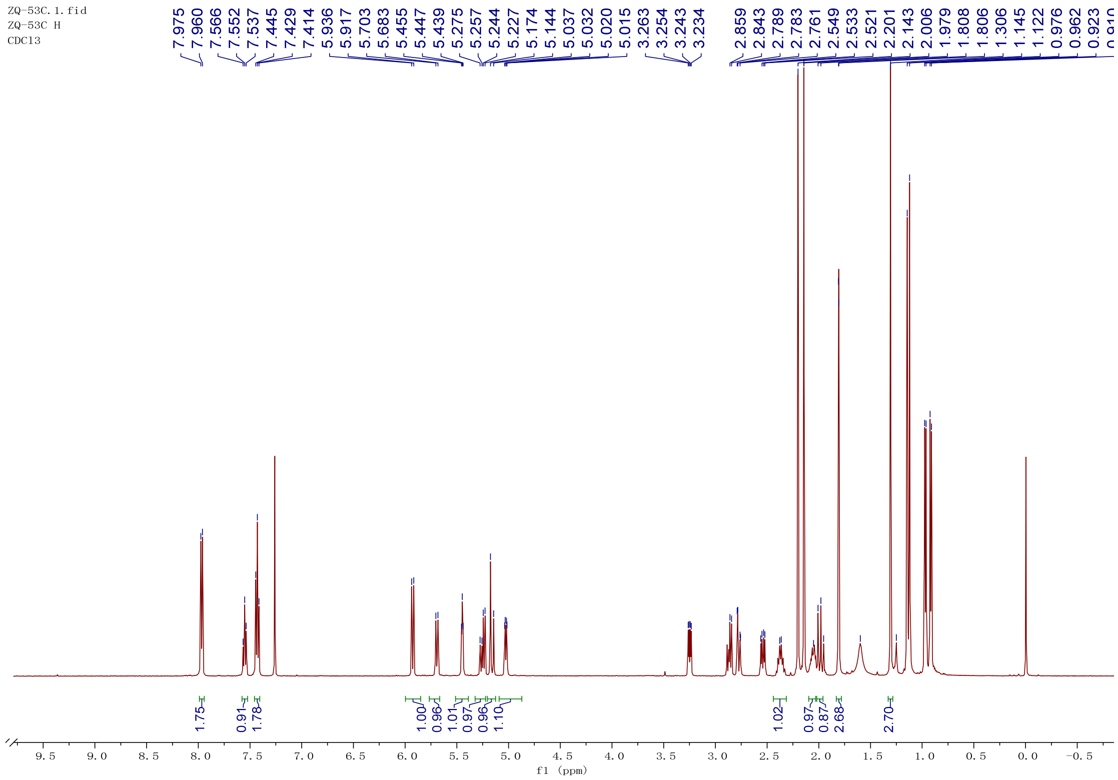

Figure S37 ^{13}C NMR spectrum of 6 (125 MHz, CDCl$_3$)

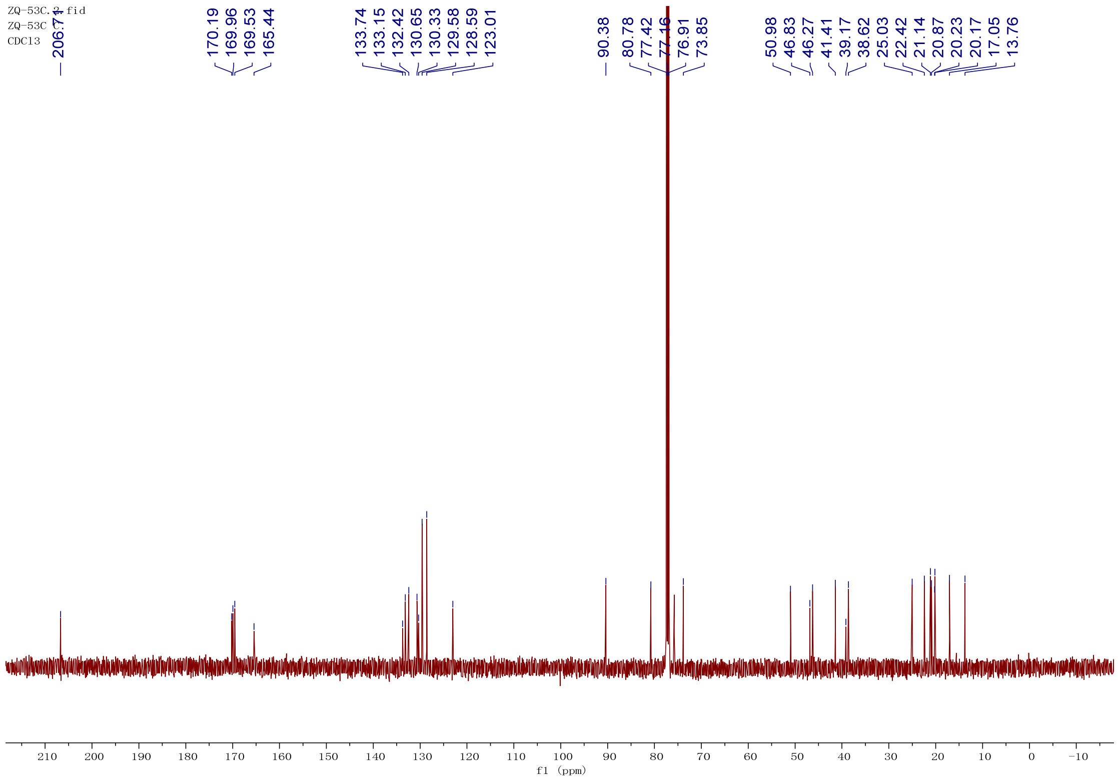

Figure S38 HSQC spectrum of 6

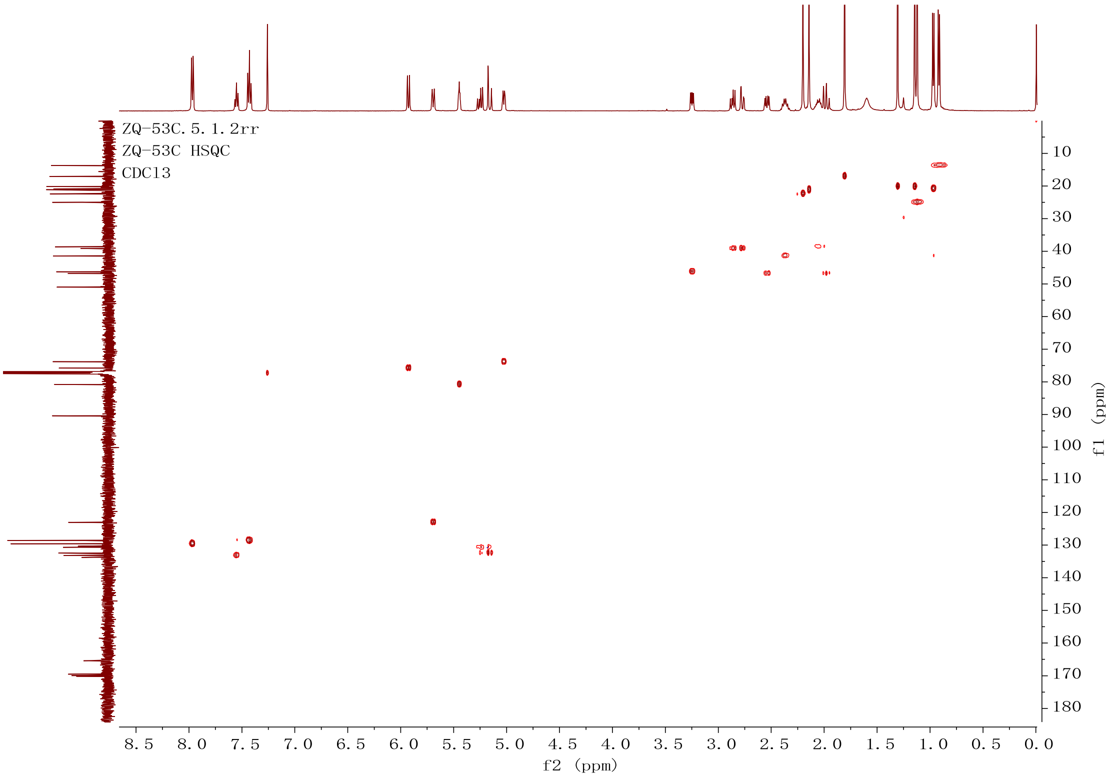

Figure S39 ^{1}H-^{1}H COSY spectrum of 6

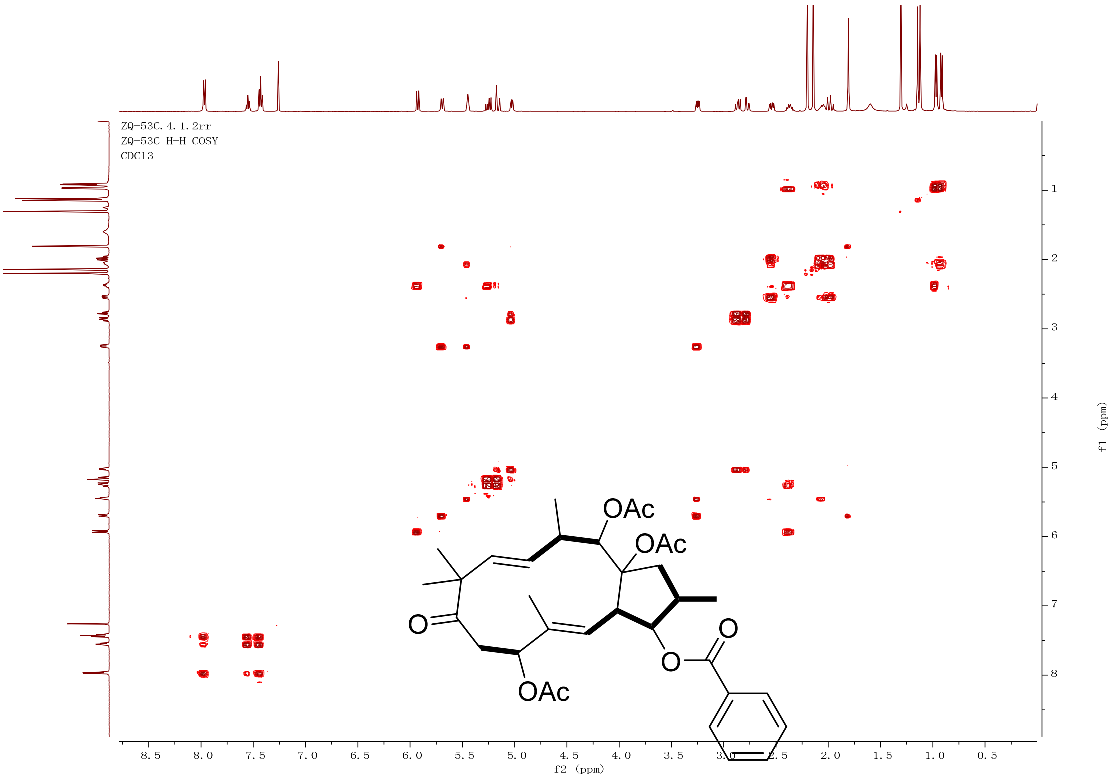

Figure S40 HMBC spectrum of 6

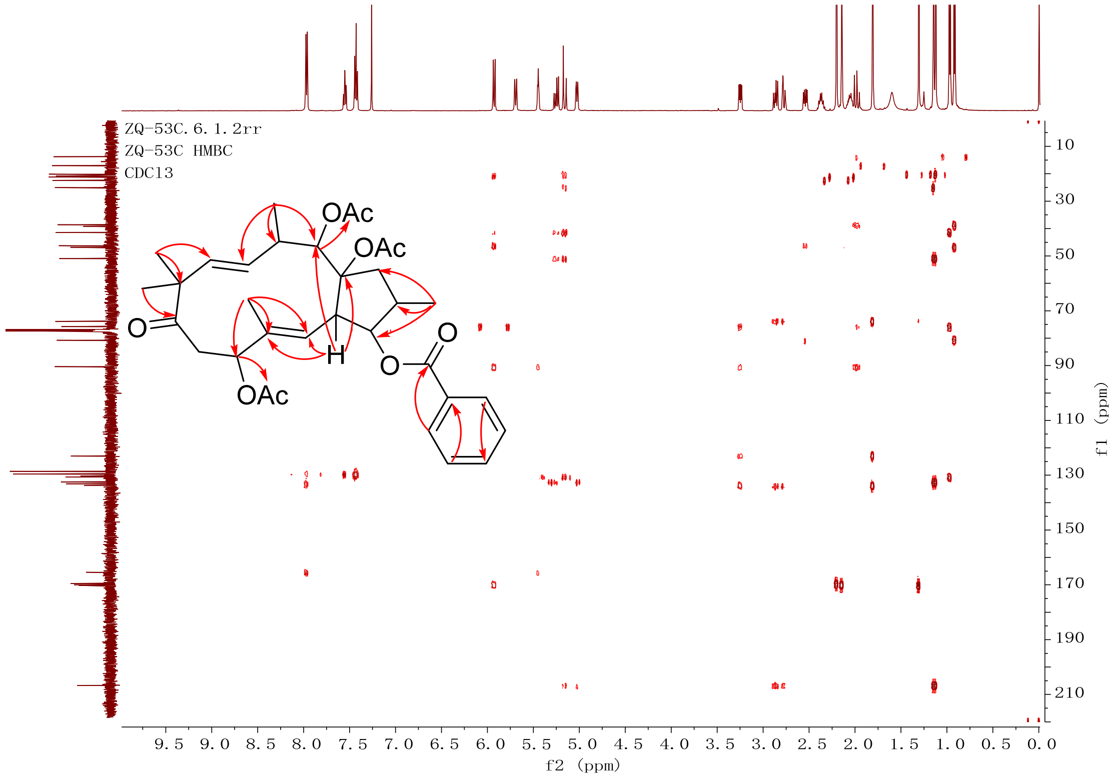

Figure S41 ROESY spectrum of 6

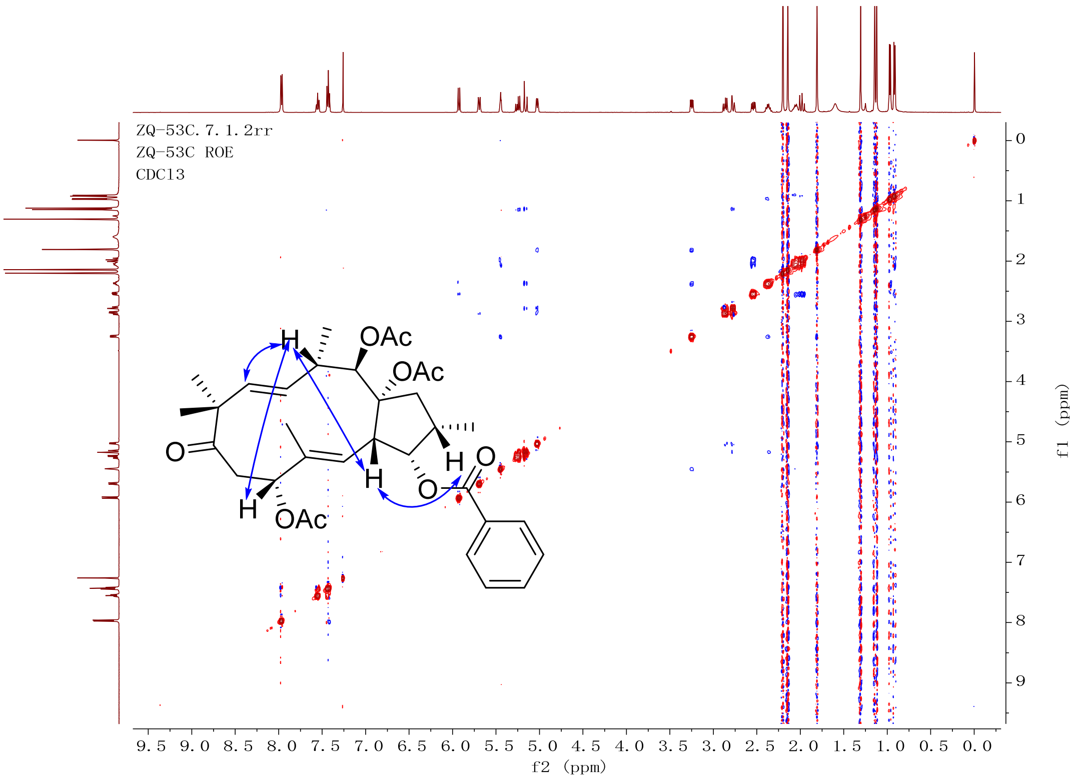

Figure S42 HRESIMS report of 6

Display Report

Analysis Info

Analysis Name	D:\Data\GJH\20220309-ZZZ\N-ZQ-53C.d	Acquisition Date	3/9/2022 4:33:15 PM
Method	tune_pos_standard_20141031.m	Operator	Demo User
Sample Name	N-ZQ-53C	Instrument	maXis HD 1820881.21303
Comment			

Acquisition Parameter

Source Type	ESI	Ion Polarity	Positive	Set Nebulizer	0.3 Bar
Focus	Active	Set Capillary	3500 V	Set Dry Heater	200 °C
Scan Begin	50 m/z	Set End Plate Offset	-500 V	Set Dry Gas	4.0 l/min
Scan End	3000 m/z	Set Charging Voltage	2000 V	Set Divert Valve	Waste
		Set Corona	0 nA	Set APCI Heater	0 °C

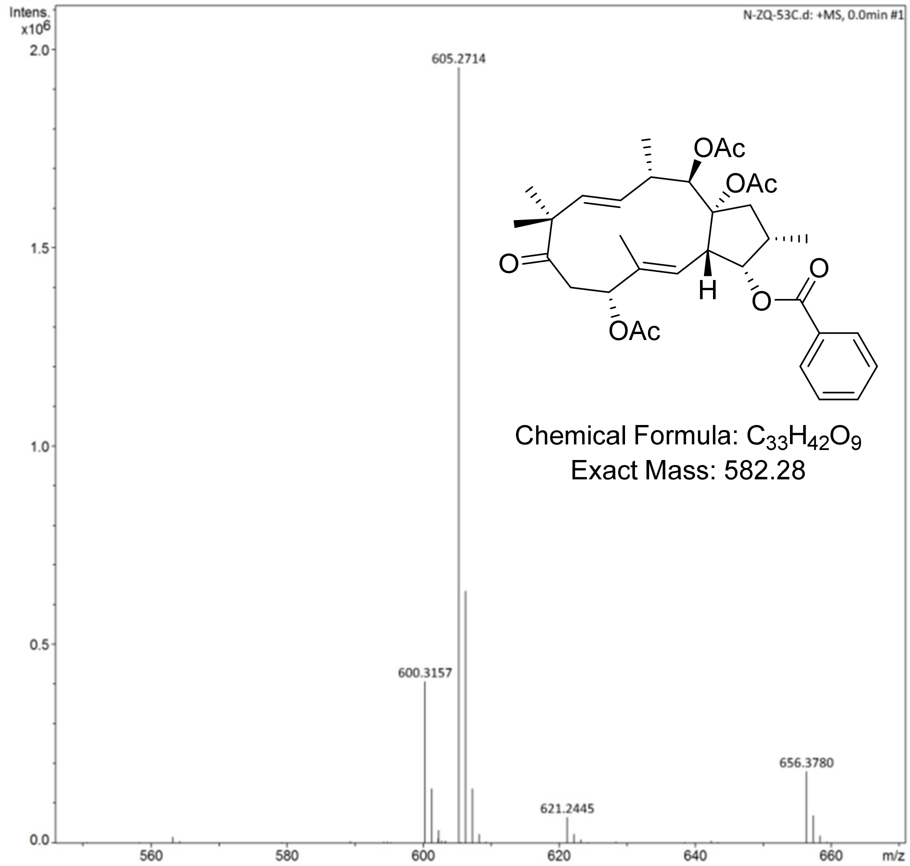

N-ZQ-53C.d

Bruker Compass DataAnalysis 4.4 printed: 3/10/2022 10:28:20 AM by: demo Page 1 of 1

Figure S43 ^{1}H NMR spectrum of 7 (500 MHz, $CDCl_3$)

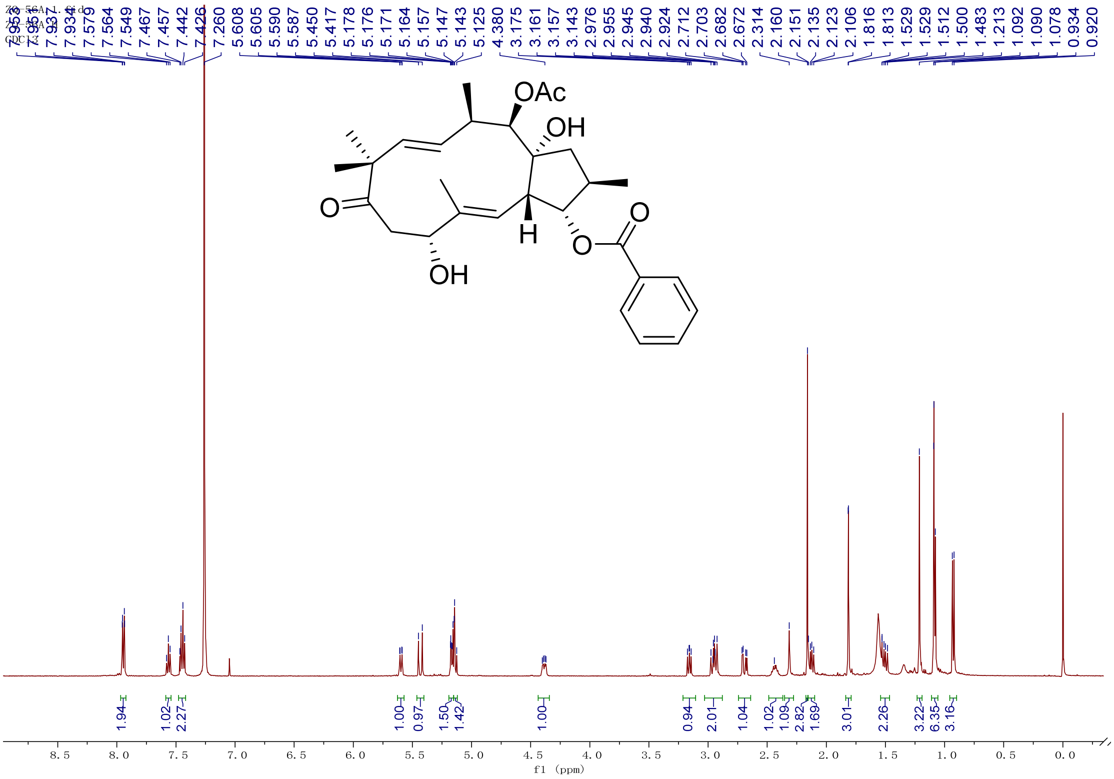

Figure S44 ^{13}C NMR spectrum of 7 (125 MHz, $CDCl_3$)

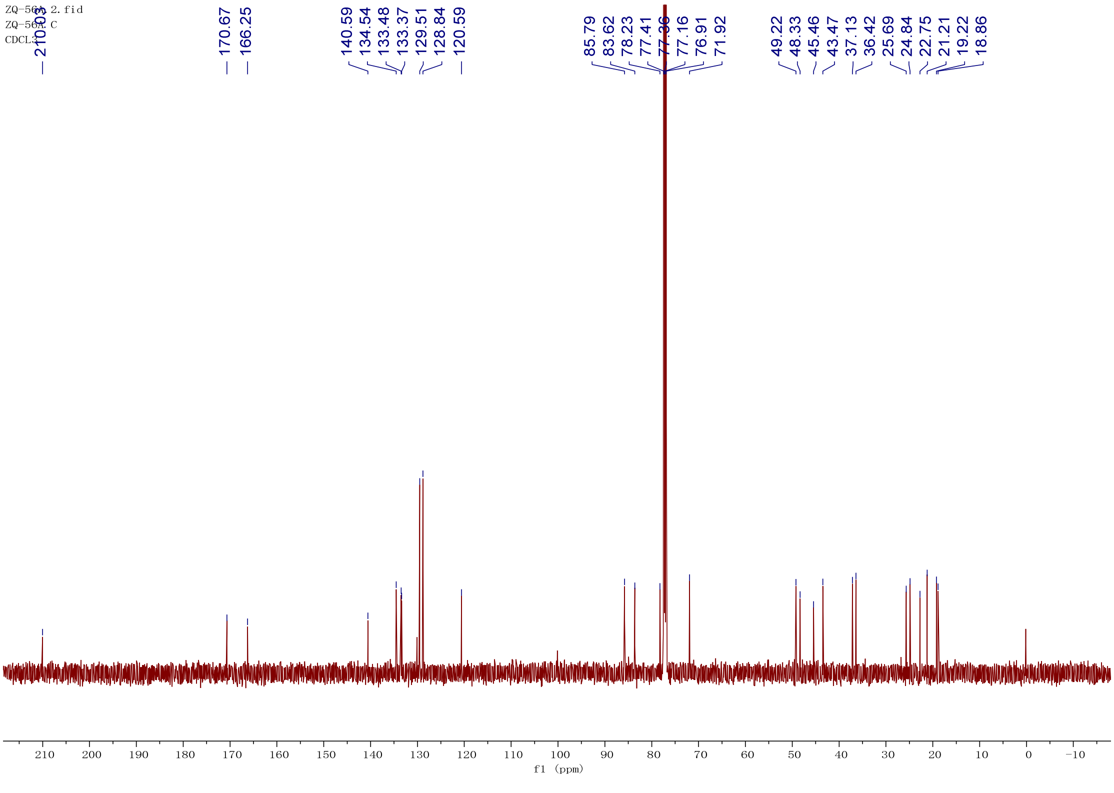

Figure S45 HSQC spectrum of 7

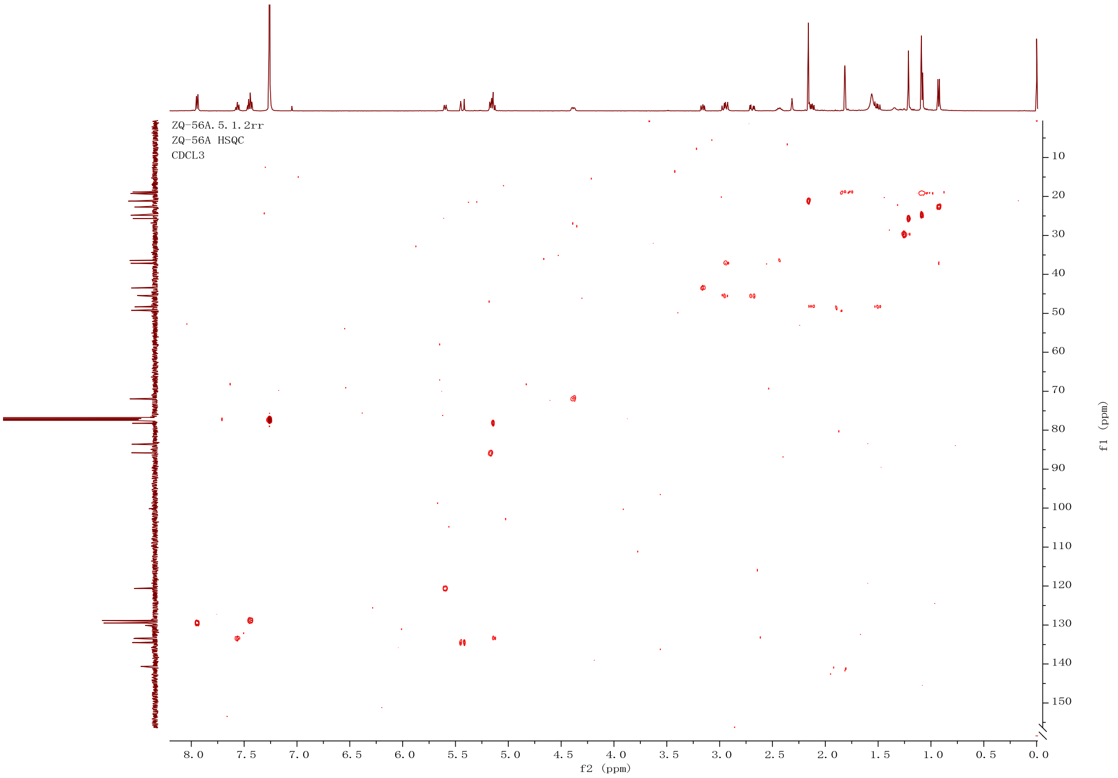

Figure S46 ^{1}H-^{1}H COSY spectrum of 7

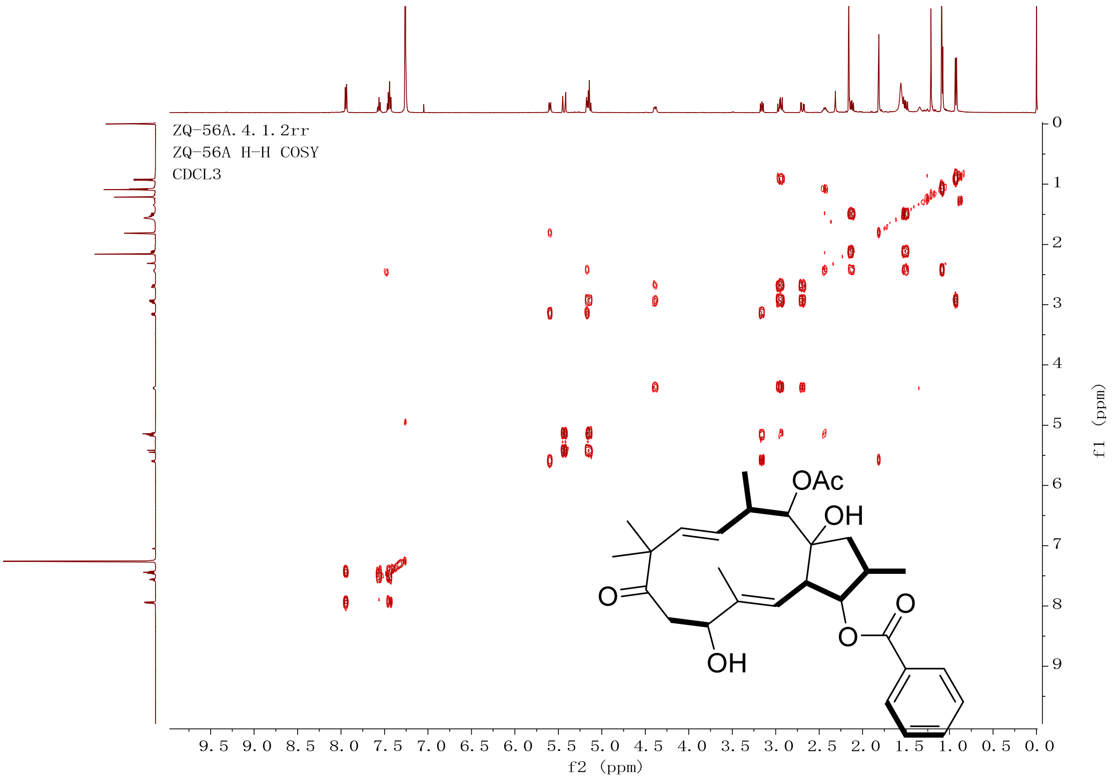

Figure S47 HMBC spectrum of 7

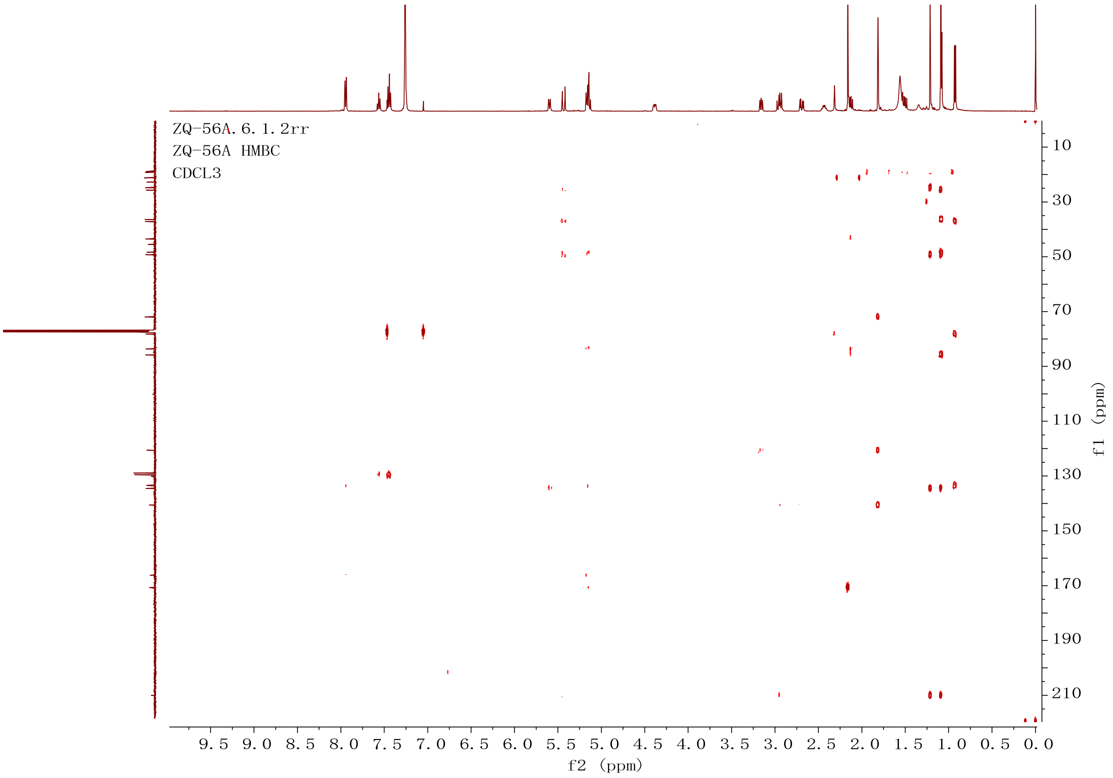

Figure S48 ROESY spectrum of 7

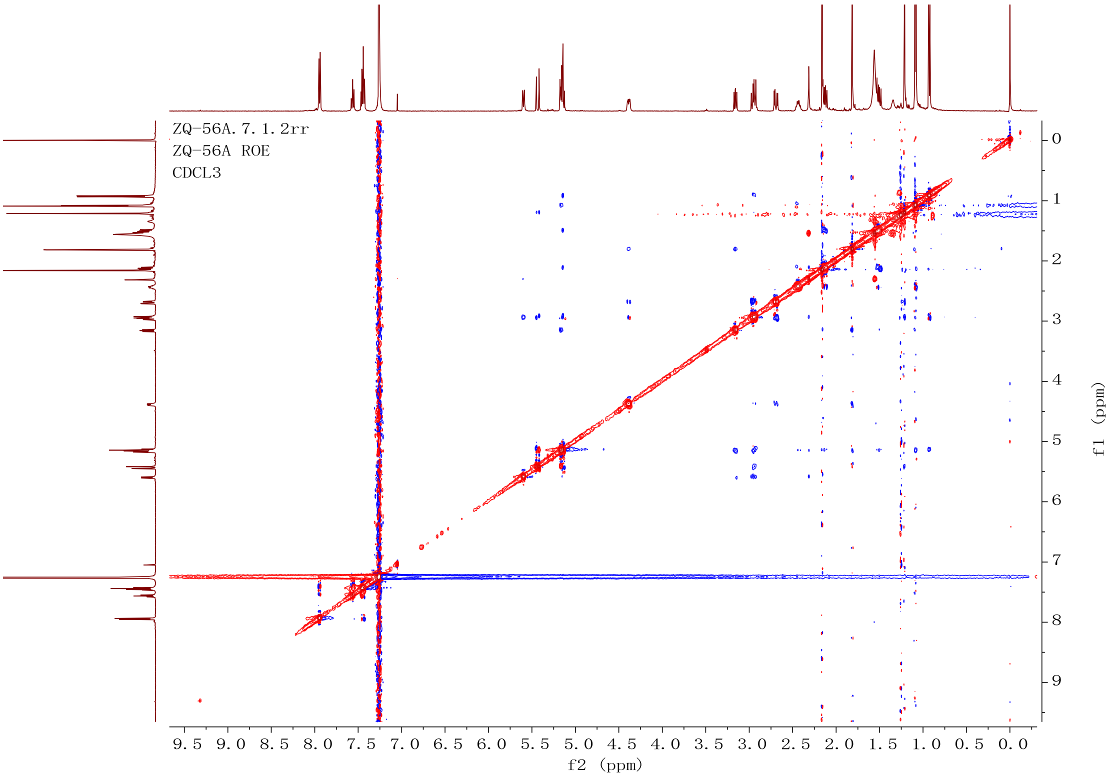

Figure S49 HRESIMS report of 7

Generic Display Report

**Analysis Info**		Acquisition Date	3/7/2023 11:38:46 AM
Analysis Name	D:\Data\GJH\ZZZ\ZQ-56A.d		
Method	tune_pos_standard_20141031.m	Operator	Demo User
Sample Name	ZQ-56A	Instrument	maXis HD
Comment			

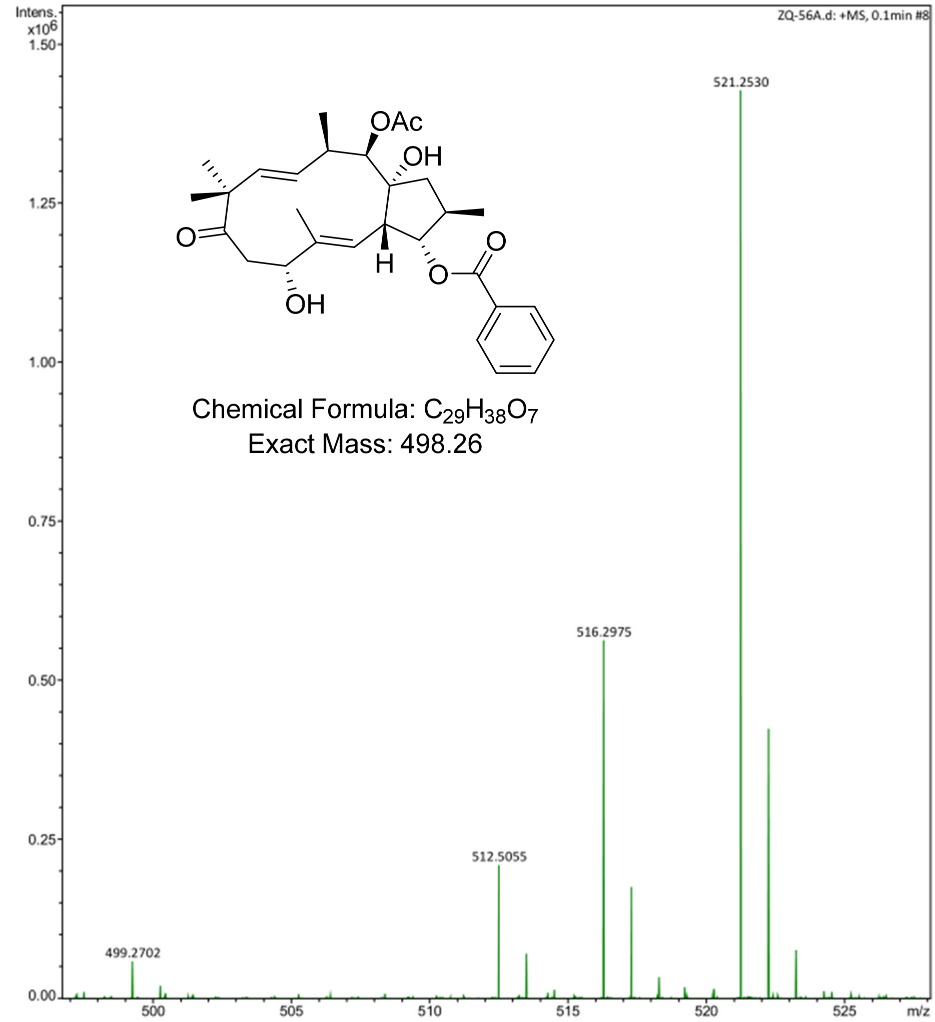

Bruker Compass DataAnalysis 4.4 printed: 3/31/2023 11:34:25 AM by: demo Page 1 of 1

Figure S46 ^{1}H NMR spectrum of 13 (500 MHz, $CDCl_3$)

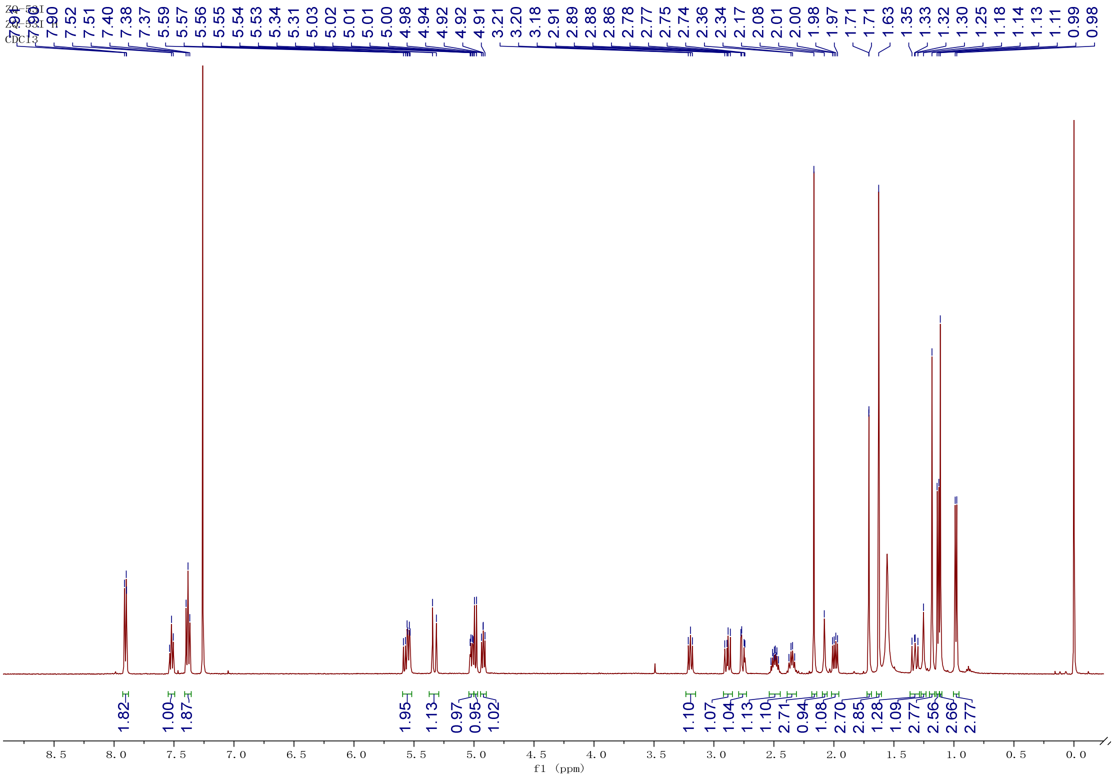

Figure S47 ^{13}C NMR spectrum of 13 (125 MHz, $CDCl_3$)

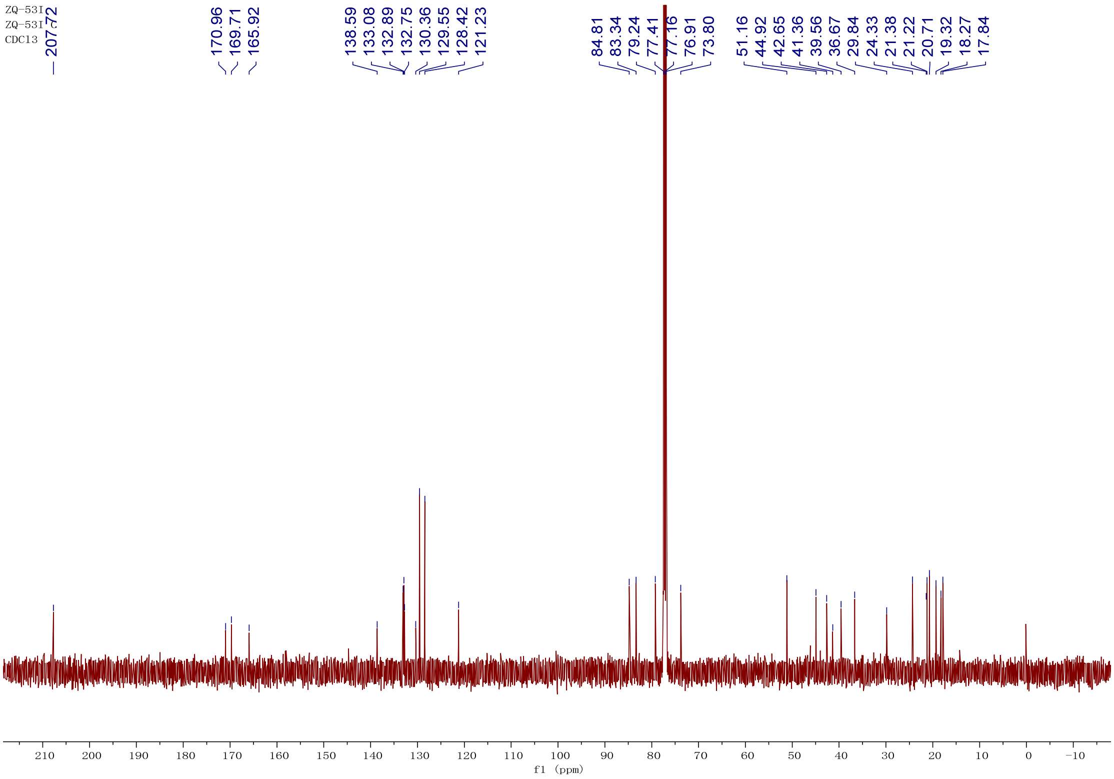

Figure S48 ^{1}H NMR spectrum of 17 (500 MHz, $CDCl_3$)

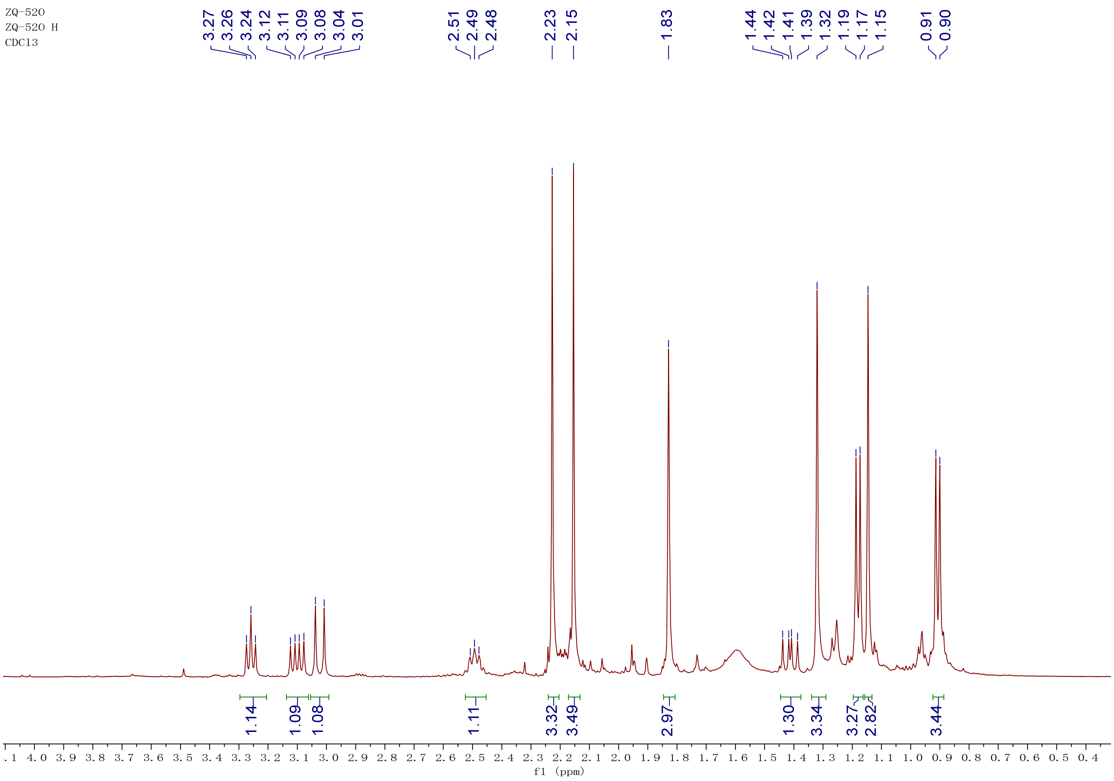

Figure S49 ^{13}C NMR spectrum of 17 (125 MHz, $CDCl_3$)

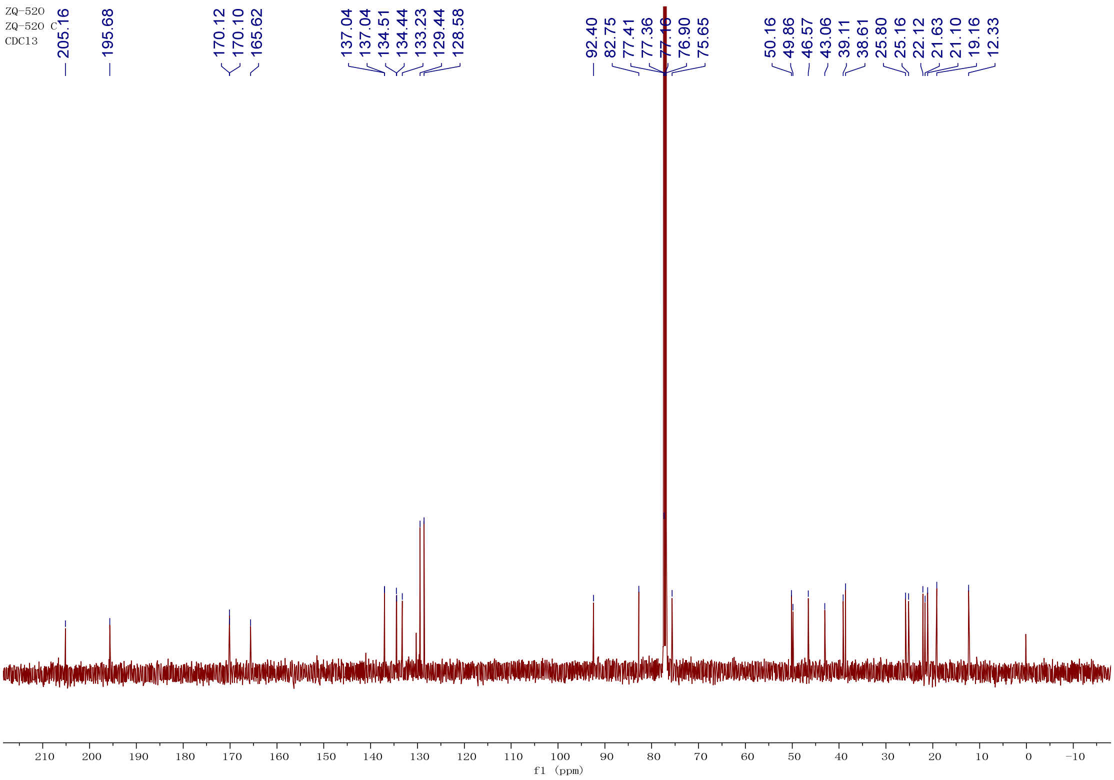

Figure S50 HSQC spectrum of 17

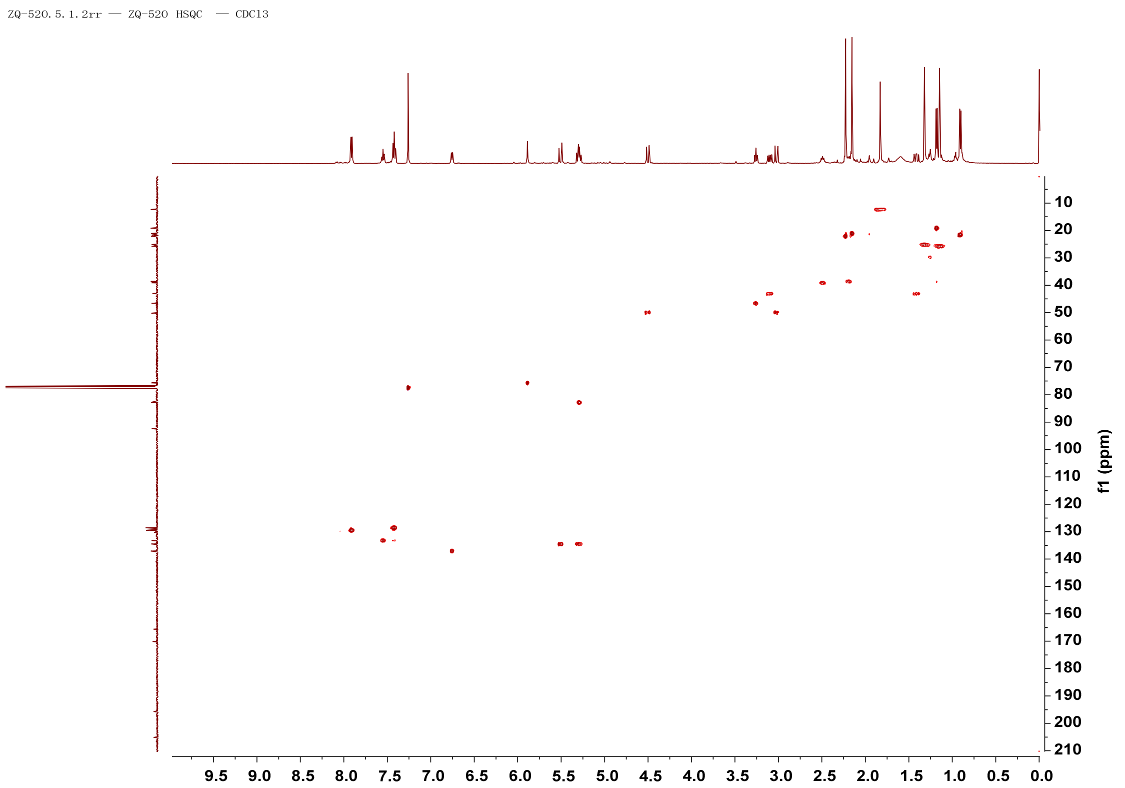

Figure S51 ^{1}H NMR spectrum of 20 (500 MHz, $CDCl_3$)

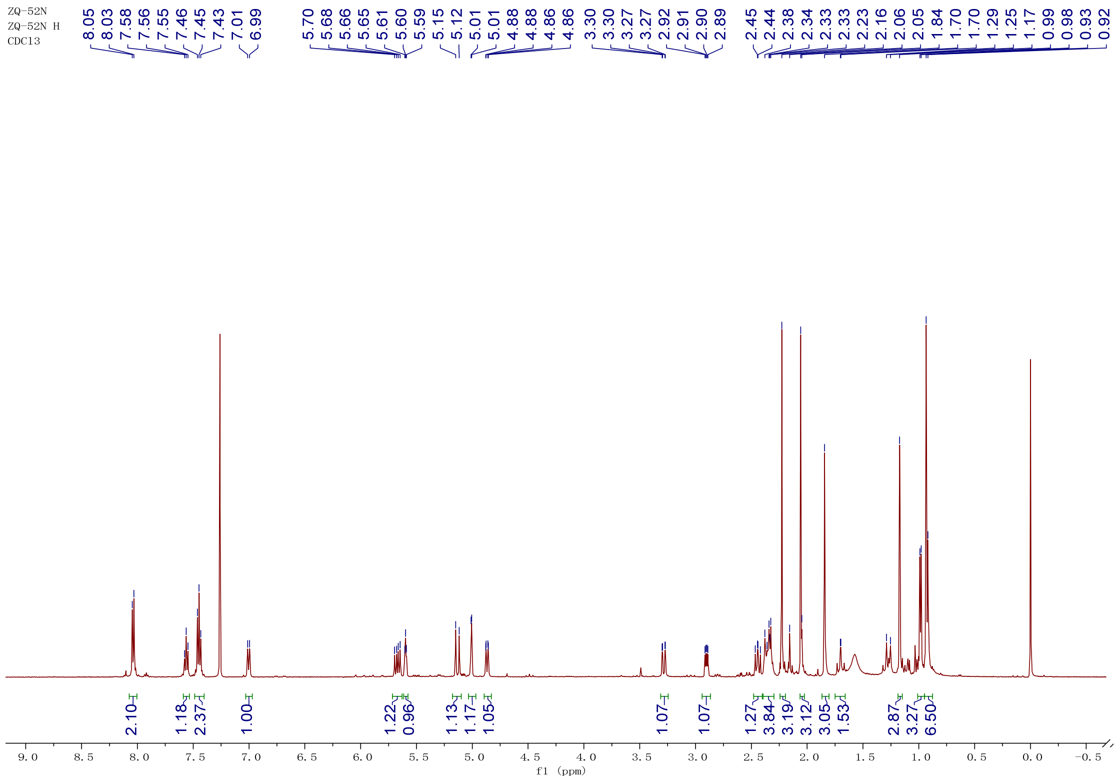

Figure S52 ^{13}C NMR spectrum of 20 (125 MHz, $CDCl_3$)

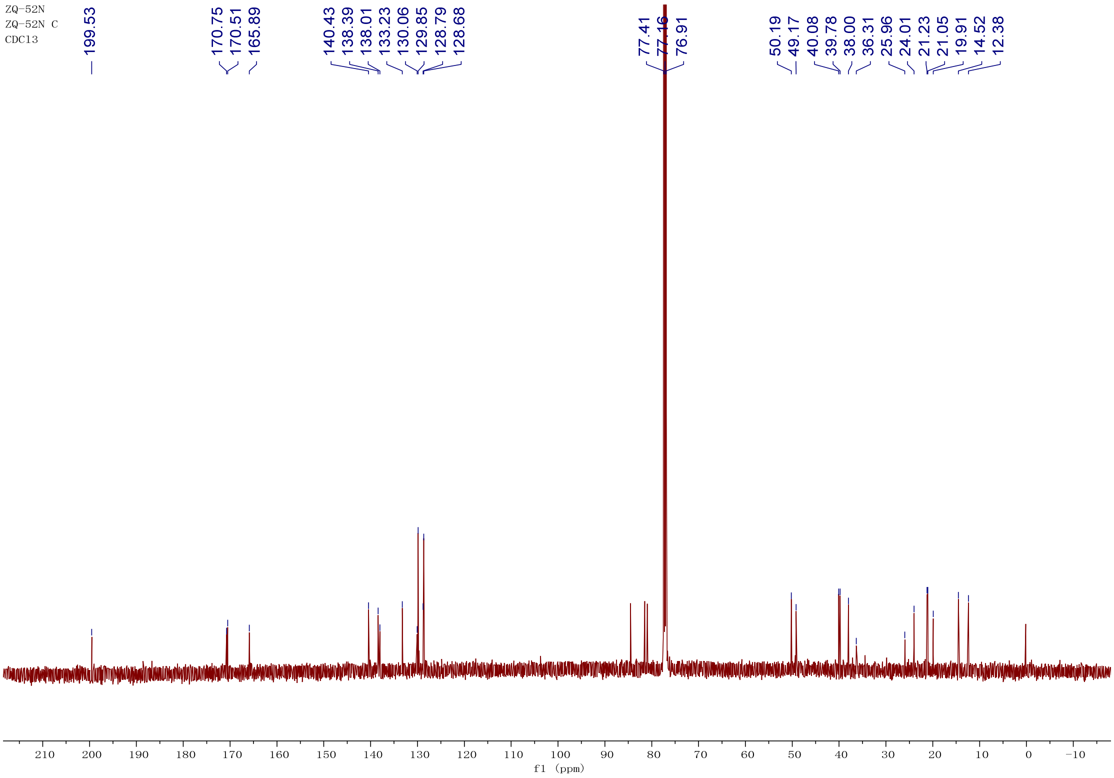

Figure S53 HSQC spectrum of 20

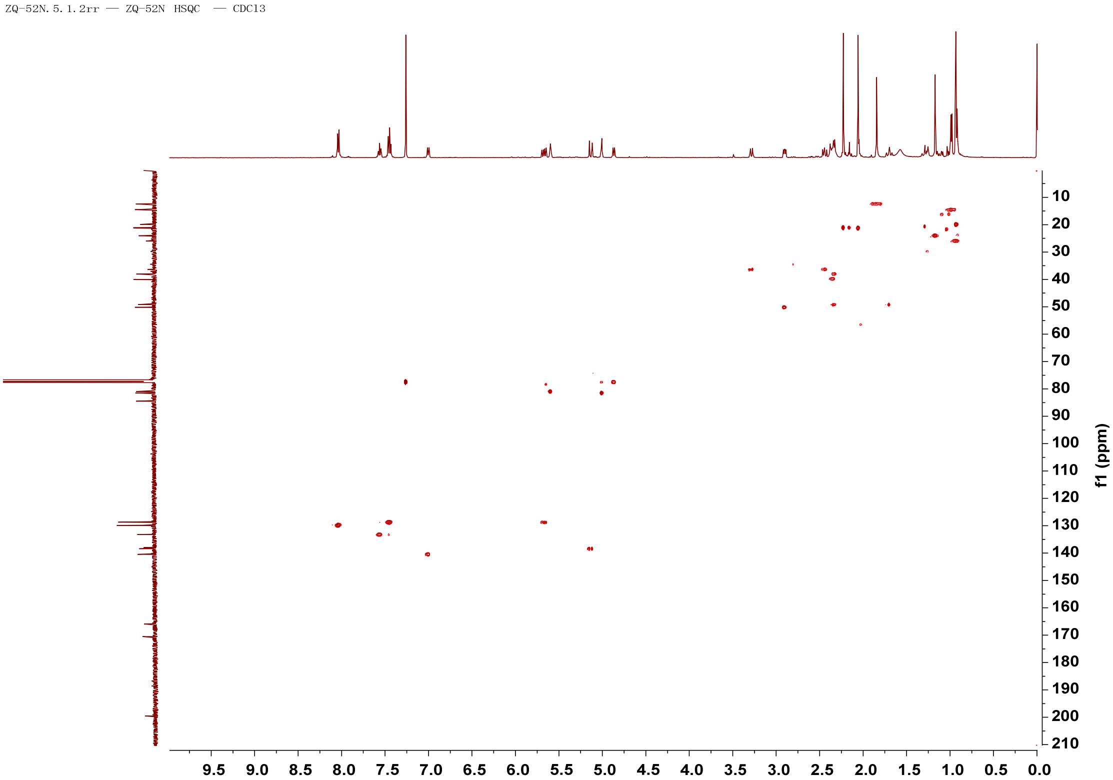

2. Computational details for 1 and 2

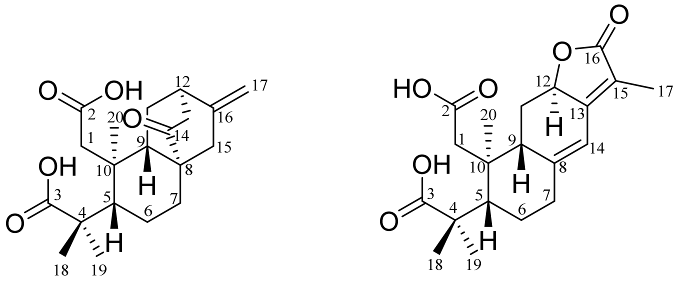

1 **2**

Conformation search based on molecular mechanics with MMFF94s force fields were performed for **1** and **2** each affording seven and six stable conformers with distributions higher than 5%[2,3], respectively. All these conformers were further optimized by the density functional theory method at the B3LYP/6-31G(d) level in Gaussian 09 program package[3], leading to six conformers for **1** and four conformers for **2** within a 3 kcal/mol energy threshold from global minimum, respectively (Tables S2 and S3). Conformers were subjected to theoretical calculation of ECD using time-dependent density functional theory (TDDFT) at B3LYP/6-31G(d) level with CPCM model in methanol based on B3LYP/6-31G(d) optimized conformers. The calculated ECD curves for **1** and **2** were weighted using SpecDis[4].

Table S2. Energy analysis for conformers of **1** at B3LYP/6-31G(d) level in the gas phase

Specie	Gibbs free energy	*ΔE* (kcal/mol)	P_E%
**1-a**	-1154.702992	1.62	4.3
**1-b**	-1154.705568	0.00	66.4
**1-c**	-1154.702063	2.20	1.6
**1-d**	-1154.703669	1.19	8.9
**1-e**	-1154.704035	0.96	13.1
**1-f**	-1154.703232	1.47	5.6
**1-g**	-1154.699622	3.73	0

Table S3. Energy analysis for conformers of **2** at B3LYP/6-31G(d) level in the gas phase

Specie	Gibbs free energy	*ΔE* (kcal/mol)	$P_E\%$
**2-a**	-1228.762974	1.40	6.7
**2-b**	-1228.763845	0.86	17.0
**2-c**	-1228.759925	3.32	0.3
**2-d**	-1228.765210	0.00	72.1
**2-e**	-1228.762444	1.74	3.8
**2-f**	-1228.759062	3.86	0.1

3. Cytokine Analysis by ELISA of Induced T cells

Table S4. Concentrations of IFN-γ/IL-2/IL-17A in different groups

Groups	*c* (μM)	Concentration (pg/mL)		
		IFN-γ	IL-2	IL-17A
**M**		22654.9±1278.5	2638.4±442.2	3395.2±80.8
**7**	2.5	11984.4±390.1****	1340.6±370.6**	2027.9±898.5 ns
**7**	1	10806.1±921.6****	1469.6±510.2**	2262.5±784.7 ns
**21**	2.5	11601.4±646.7****	1280.3±139.9**	2150.3±430.0 ns
**21**	1	15945.9±695.9****	1774.7±540.0 ns	2992.9±684.7*
**Dex**	0.05	13914.5±1764.1****	1387.5±294.7**	1557.2±432.5****
**Y**		309.4±29.5****	untested****	untested****

Note: *vs* M, **** $P < 0.0001$, ***$P < 0.001$, **$P < 0.01$, *$P < 0.05$

4. EdU results for 7 and 21

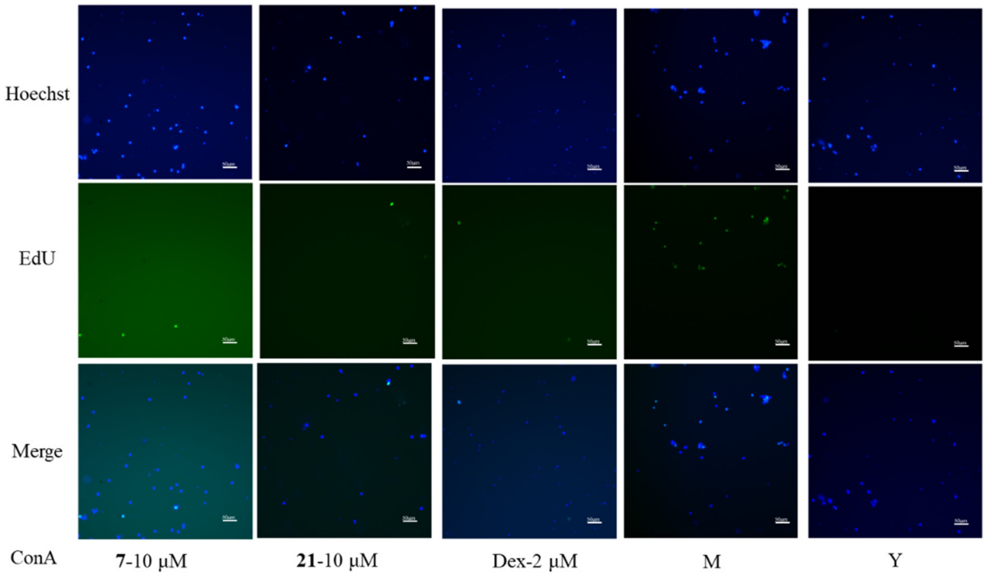

Figure S54. Inhibitory activities of **7** and **21** against induced T cells (ConA) measured by EdU methods.

References:

(1) Goto, H., Osawa, E., 1989. Corner flapping: a simple and fast algorithm for exhaustive generation of ring conformations. *J. Am. Chem. Soc. 111*, 8950–8951.

(2) Goto, H., Osawa, E., 1993. *J. Chem. Soc., Perkin Trans. 2*, 187–198.

(3) Frisch, M. J., Trucks, G. W., Schlegel, H. B., Scuseria, G. E., Robb, M. A., Cheeseman, J. R., Scalmani, G., Barone, V., Mennucci,B., Petersson, G. A., Nakatsuji, H., Caricato, M., Li, X., Hratchian, H.P., Izmaylov, A. F., Bloino, J., Zheng, G., Sonnenberg, J. L., Hada, M., Fukuda, R., Hasegawa, J., Ishida, M., Nakajima, T., Honda, Y., Kitao,O., Nakai, H., Vreven, T., Montgomery, J. A., Ogliaro, F., Bearpark,M., Heyd, J. J., Brothers, E., Kudin, K. N., Staroverov, V. N., Keith, T.,Kobayashi, R., Normand, J., Raghavachari, K., Rendell, A., Burant, J.C., Iyengar, S. S., Tomasi, J., Cossi, M., Rega, N., Millam, J. M., Klene,M., Knox, J. E. J., Cross, B., Bakken, V., Adamo, C., Jaramillo, J.,Gomperts, R., Stratmann, R. E., Yazyev, O., Austin, A. J., Cammi, R.,Pomelli, C., Ochterski, J. W., Martin, R. L., Morokuma, K.,Zakrzewski, V. G., Voth, G. A., Salvador, P., Dannenberg, J. J.,Dapprich, S., Daniels, A. D., Farkas, O., Foresman, J. B., Ortiz, J. V.,Cioslowski, J., Fox, D. J. 2016. Gaussian, Inc., Wallingford CT.

(4) Bruhn, T., Schaumlöffel, A., Hemberger, Y., Bringmann, G., 2012. *Spec Dis*, version 1.71, University of Würzburg, Germany,
